# Declining subjective well-being disparities concurrent with urbanization in China

**DOI:** 10.1093/nsr/nwaf362

**Published:** 2025-08-29

**Authors:** Ranhao Sun, Wenning Li, Hongbin He, Song Leng, Jianquan Cheng, Xiaojun Yang, Zhaowu Yu, Liding Chen

**Affiliations:** State Key Laboratory of Regional and Urban Ecology, Research Center for Eco-Environmental Sciences, Chinese Academy of Sciences, Beijing 100085, China; University of Chinese Academy of Sciences, Beijing 100049, China; State Key Laboratory of Regional and Urban Ecology, Research Center for Eco-Environmental Sciences, Chinese Academy of Sciences, Beijing 100085, China; Beidou Application Development Research Institute, Beijing 100080, China; State Key Laboratory of Regional and Urban Ecology, Research Center for Eco-Environmental Sciences, Chinese Academy of Sciences, Beijing 100085, China; University of Chinese Academy of Sciences, Beijing 100049, China; State Key Laboratory of Regional and Urban Ecology, Research Center for Eco-Environmental Sciences, Chinese Academy of Sciences, Beijing 100085, China; Department of Natural Sciences, Manchester Metropolitan University, Manchester M1 5GD, UK; Department of Geography, Florida State University, Tallahassee, FL 32306, USA; Department of Environmental Science and Engineering, Fudan University, Shanghai 200438, China; State Key Laboratory of Regional and Urban Ecology, Research Center for Eco-Environmental Sciences, Chinese Academy of Sciences, Beijing 100085, China; University of Chinese Academy of Sciences, Beijing 100049, China

**Keywords:** urbanization, inequality, subjective well-being, urban environment, healthy living

## Abstract

While urbanization has significantly reshaped cities globally, its complex multidimensional impacts on subjective well-being (SWB) remain poorly understood. We develop an integrated ecological and social framework using 2.92 million street view images and assess its relationship to urban residents’ SWB derived from 5.3 million social media posts across 107 Chinese cities. Our study reveals a notable decline in SWB disparities, with a significant increase in the lowest SWB class and a corresponding decrease in the highest class. Urban ecological factors, such as vegetation coverage and street ecological index, are positively correlated with SWB in lower well-being populations but negatively associated with SWB in higher well-being groups. Conversely, urban social and economic determinants, including population density and service accessibility, exhibit opposite trends, revealing the multifaceted and complicated mechanisms through which urbanization processes influence residents’ SWB. This research underscores the critical role of urban environments in mitigating SWB disparities and suggests that policies enhancing environmental quality and social equity are crucial for fostering healthier, more equitable cities.

## Teaser

Urbanization exerts opposing effects on populations at different levels of well-being through ecological and socioeconomic pathways, thereby significantly narrowing the gap in residents' subjective well-being.

## INTRODUCTION

Global urbanization underscores disparities in urban economy and infrastructure [1,2]. However, the impact of urbanization on subjective well-being (SWB), which includes emotional responses and cognitive evaluations of life quality, remains ambiguous [3,4]. Urbanization profoundly reshapes SWB through multiple pathways: transforming economic opportunities, reconfiguring social support networks, altering environmental conditions, and restructuring access to essential services across the urban landscape [5]. The urban transition fundamentally reconfigures social support systems and simultaneously intensifies economic stratification that underpins residents’ SWB. Despite these insights, the persistent challenge lies in constructing cities that are inclusive, safe and resilient, aligning with the United Nations Sustainable Development Goals (SDGs) [6,7].

Urbanization brings about significant benefits for SWB, offering enhanced opportunities for employment, education and leisure [8,9]. However, the fast-paced nature of urban life introduces considerable social stressors [10] and elements of insecurity [11], which can negatively impact SWB. Furthermore, phenomena such as hedonic adaptation and social contrast, inherent to urbanization processes, can significantly reduce SWB [12]. Urbanization not only transforms social systems and influences human well-being, but it also reshapes urban landscapes and has significant impacts on environmental quality [13,14]. While natural landscapes are known to bolster SWB [15,16], urban dwellers frequently face stressors associated with noise, pollution and heatwaves [17–19]. Alarmingly, a substantial portion of the urban population struggles with severe psychological distress and depressive disorders [20,21]. Economic and social inequalities significantly mediate the relationship between urbanization and SWB by shaping access to resources, opportunities and social cohesion in urban environments [22–24]. A recent study examines spatial inequality across Chinese cities, revealing how socioeconomic segregation creates disparate access patterns to critical well-being resources [25]. Physical urban configurations increasingly separate population groups along socioeconomic lines, resulting in stratified accessibility to essential amenities including healthcare facilities, educational institutions, cultural spaces and high-quality green areas. These structured disparities in environmental resources systematically shape residents’ daily lived experiences and create persistent well-being disadvantages for marginalized urban populations. A nuanced understanding of how rapid urbanization reshapes social and ecological systems—and how these changes influence SWB—is essential for designing urban environments that promote urban sustainability. Nevertheless, the complexity of this interaction is further intensified by evidence suggesting that the evolution of social and ecological systems may vary depending upon the different stages of urban development [26–28]. The complex and multifaceted relationship between urbanization and SWB underscores the need for a nuanced understanding of how social and ecological factors shape SWB across the urbanization gradient.

In the context of China, the past three decades have witnessed an unparalleled and heterogeneous wave of urbanization [29–31], resulting in significant shifts in socioeconomic compositions and ecological infrastructures, with implications for public health [32]. Research has highlighted a notable increase in the aggregate levels of SWB among the Chinese people from 2013 to 2017 [33], with rural areas experiencing a particularly pronounced increase in SWB [34]. However, current studies rely on cross-sectional or aggregate data to characterize SWB, limiting the ability to capture individual experiences and temporal fluctuations. This limitation leads to a significant gap in understanding the multifarious effects of urbanization on SWB and the integral roles played by social and ecological systems. Addressing this gap necessitates the adoption of longitudinal study designs and individual-level data analysis to unravel the nuanced trajectories of SWB amid the urbanization process. The burgeoning of big data and deep-learning technology offers unprecedented opportunities to elucidate the intricate profiles of urban environments and their inhabitants [35].

In this study, we leverage recent advancements to examine how various socioeconomic and ecological factors influence SWB in the context of rapid urbanization. Initially, we use nighttime light (NTL) imagery to delineate annual urban boundaries from 1992 to 2021, depicting the urbanization gradients over the past three-decade period. We then employ deep learning-based language algorithms to analyze SWB using a dataset of 5.31 million posts from the Chinese microblogging platform Weibo in 2021. Additionally, we develop a street ecological index (SE) using 2.92 million street view images in 2021 analyzed by a deep learning-based algorithm, the pre-trained DeepLabv3 model. We explore SWB variations across urbanization gradients, examining factors like the SE, normalized difference vegetation index (NDVI), air temperature (AT), road density (RD), population density (POP), public-service facility density (PS) and gross domestic product (GDP). Our analysis tracks geographical dynamics of SWB and highlights how these factors influence SWB across urbanization gradients.

## RESULTS

### Socioeconomic and ecological changes over urbanization gradients

**Figure 1.**
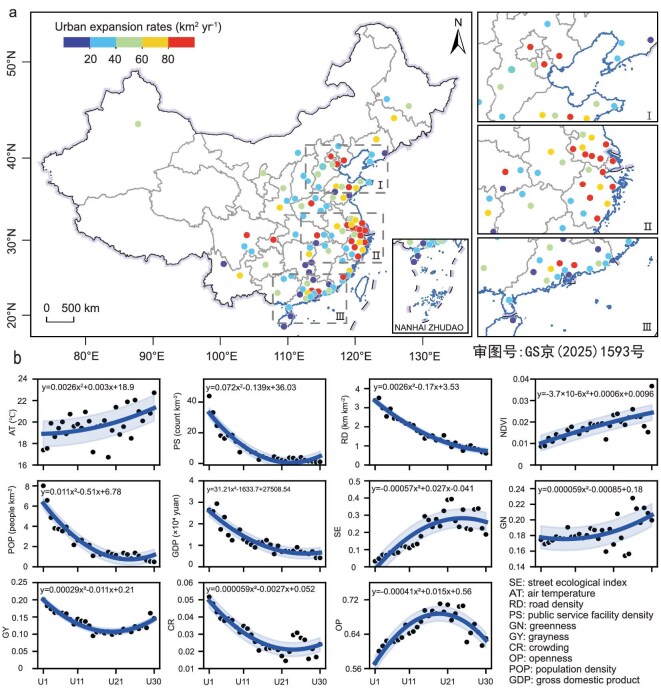
Social and ecological changes during China's urbanization. (a) Urban expansion rates in 107 Chinese cities. (b) Variations in 11 socioecological factors over urbanization gradients from U1 to U30, representing the urban boundary from 1992 to 2021.

China has experienced rapid urbanization over the past 30 years. By 2021, the mean area of 107 cities had reached 1990.5 km^2^, with individual cities ranging from 344.4 to 6582.3 km^2^ (Fig. 1a). In contrast, the mean city area in 1992 was 196.4 km^2^, with a range of 0.5 to 1418.6 km^2^. Over the 30 year period, the collective area of these cities expanded more than 10-fold, growing from 2.0 × 10^4^ to 21.2 × 10^4^ km^2^. The average rate of city expansion was 63.2 km^2^ per year, with individual cities expanding from 10.8 to 215.7 km^2^ annually (Fig. S1; Table S1). Coastal cities in the eastern and southern China exhibited significantly higher growth rates compared to other regions. We analyzed urban streetscape data using the pre-trained DeepLabv3 model to derive four indices: greenness (GN), grayness (GY), openness (OP) and crowdedness (CR). Along with the urbanization gradients from urban to rural regions, GN and OP have notably risen, whereas GY and CR have markedly declined (Fig. S2). The SE, a metric by combining the above four indices reflecting the quality of urban green infrastructure, experienced rapid growth in urban regions, followed by relative stability in rural regions. The average NDVI showed a sustained increase along the urbanization gradient, while AT exhibited a fluctuating increase. The socioeconomic indicators of RD, POP, GDP and PS exhibited similar trends, showing the transition of rapid declines to relative stability (Fig. 1b).

### Reduced SWB disparities along with urbanization

**Figure 2.**
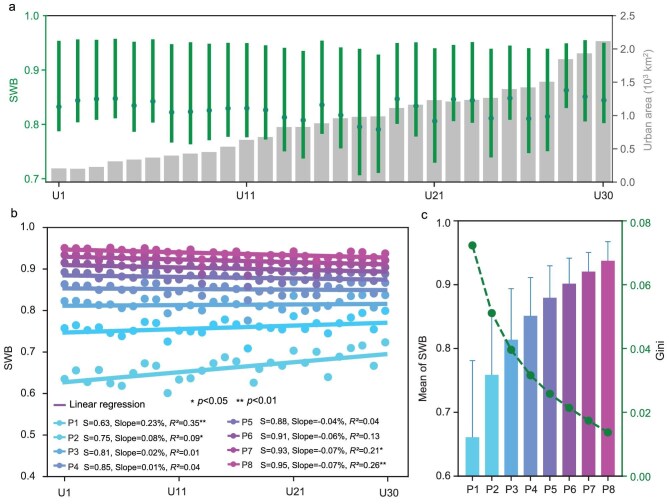
Classes and changes of SWB over urbanization gradients. (a) Spatial dynamics of SWB from U1 to U30, representing the urban boundary from 1992 to 2021. The dots and lines display the mean value, lower 25% and upper 75% quartiles of the SWB across the 107 cities in each year, and the bars are the natural urban area. (b) Variability of SWB among different SWB classes from P1 to P8. (c) Standard deviation in different levels of SWB. The bar heights showed mean value, and error bars show one standard deviation. Green dots refer to the Gini index.

Utilizing anonymized data from Weibo, a leading premier social media platform in China, this study quantifies SWB across a significant dataset, encompassing 5.31 million geotagged posts generated by 3.83 million users between September 2020 and April 2021 (Fig. S3). We employed a deep learning-based bidirectional encoder representations from transformers (BERT) algorithm to effectively extract SWB measures from this voluminous collection (Fig. 2a). Our analysis reveals that the mean SWB values across various cities not only demonstrate a relatively high baseline but also maintain stability over the urbanization gradients (Fig. S4). We stratified these SWB values into eight distinct levels, ranging from the lowest decile (P1) to the highest 80th percentile (P8) (Fig. S5; Table S2). A statistically significant uptick is noted in the SWB values within the lowest two brackets, with annual increases of 0.23% for P1 and 0.08% for P2, respectively (*P* < 0.05). Conversely, the upper two brackets exhibited a marked decline, with both P7 and P8 decreasing by 0.07% per urbanization gradient (*P* < 0.05). The intermediate SWB levels, however, do not display any significant annual variation over urbanization gradient. Notably, the changes in SWB exhibited a convergence from the 10th to the 80th percentiles, indicating a reduction in well-being inequality. There was a substantial 12% decrease in the inequality of SWB between P1 and P8, resulting in a clear change from 0.57 to 0.50 (Fig. 2b). The average SWB value across 107 cities is 0.82 ± 0.18, with incremental increases observed across all levels: P1 at 0.66 ± 0.12, P2 at 0.76 ± 0.10, P3 at 0.81 ± 0.08, P4 at 0.85 ± 0.06, P5 at 0.88 ± 0.05, P6 at 0.90 ± 0.04, P7 at 0.92 ± 0.03 and P8 at 0.94 ± 0.03 (Fig. 2c). Meanwhile, a significant decrease in Gini index of SWB was observed from P1 to P8.

### Drivers of SWB variation

**Figure 3.**
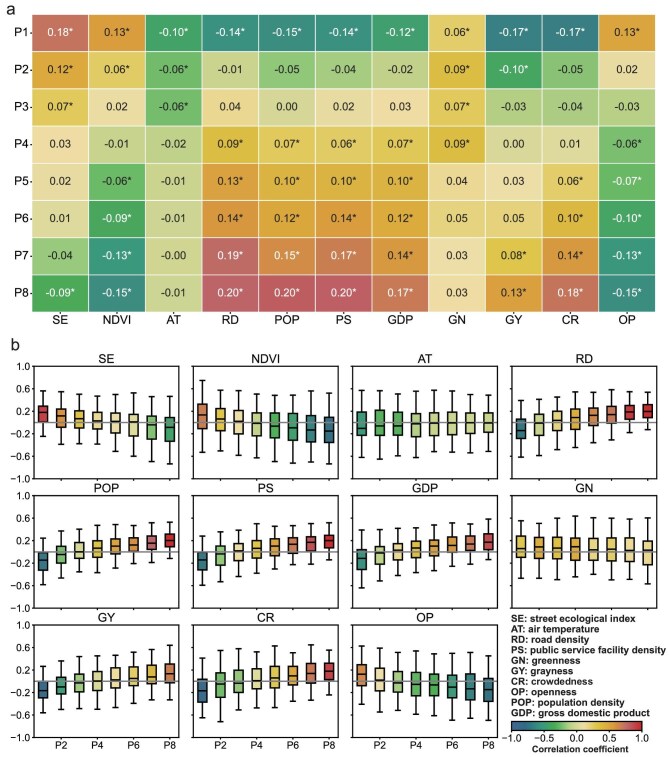
Correlation analysis between SWB levels and influencing factors. (a) Average correlation coefficients between eight distinct levels of SWB, from P1 (lowest) to P8 (highest), and influencing factors. (b) Variability in the degree to which each factor influences SWB across different levels. Boxes, intervals between the 25th and 75th percentile and median (horizontal line); whiskers, 1.5-fold interquartile range. *p < 0.05.

Our analysis elucidates a nuanced relationship between various influential factors and SWB (Fig. 3a). Notably, while AT consistently exhibits a negative correlation with SWB across all levels, the impact of other determinants varies depending on the SWB levels. Specifically, as we transition from lower to higher SWB levels, the association with the SE and NDVI weakens, whereas the correlation with PS, RD and PD becomes more pronounced. This trend suggests that ecological factors play a pivotal role in enhancing the SWB of individuals experiencing lower SWB levels, whereas social factors are more closely linked to those experiencing higher levels of SWB (Fig. 3b). Additionally, we observed a shift in the relationship between GDP and SWB, from negative in P1 to positive in P8. In contrast to satellite-derived NDVI, street image-derived GN consistently showed positive correlations with SWB across all SWB levels.

**Figure 4.**
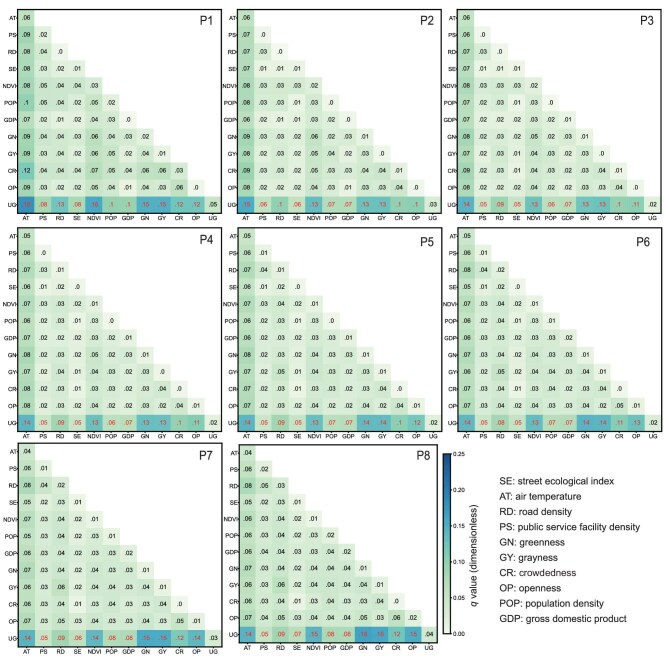
The *q* values of socioecological factor interaction among each SWB group based on Geodetector analysis. UG refers to the urbanization gradient (U1 to U30). Red numbers indicate statistically significant *q* values (*P* < 0.05), while black numbers indicate non-significant *q* values (*P* > 0.05).

We conducted additional analyses using multiple approaches, such as generalized additive models (GAMs) and Geodetector analysis, to examine the relationships between these influential factors and SWB. Results revealed insignificant relationships (*P* > 0.05) between individual factors and SWB, except when combined with urbanization gradients for all SWB levels (Fig. 4). This highlights that the mechanisms linking socioecological factors to SWB are highly complex and likely involve multiple interacting variables rather than simple direct relationships.

**Figure 5.**
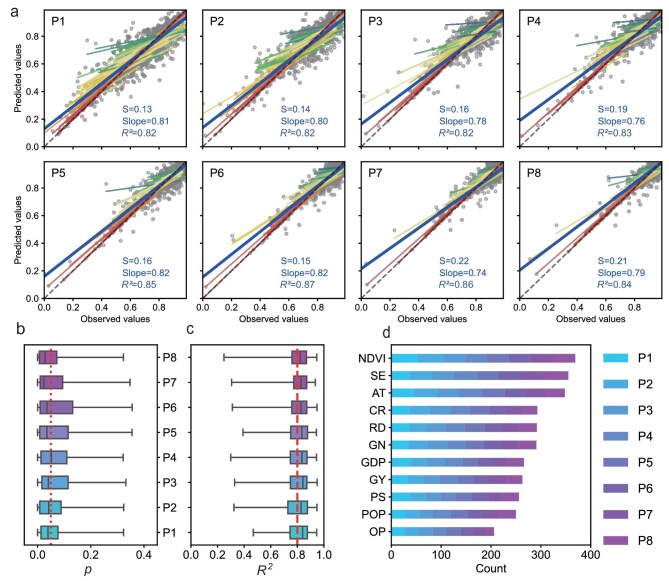
Predictive performance and indicator significance for SWB models. (a) Fit line between observed and predicted SWB on eight levels (P1–P8) across the 107 cities. The colored lines indicate individual city fitting lines and the bold blue line depicts the aggregate mean fitting line across cities. (b) Boxplot analysis of the models’ predictive accuracy, lower 25% and upper 75% quartiles of both the *R*^2^ and *p* values, with whiskers extending to 1.5-fold the interquartile range to represent variability. (c) The cumulative count of 11 influencing factors used in the model.

Utilizing a regression discontinuity design, our analysis reveals that the absolute difference (δ) between the two fitted lines at the cut-off is consistently below 0.05 across all cases (see Fig. S6). Therefore, we posit a linear relationship between the eight discrete levels of SWB and the 11 normalized key influencing factors. Employing a stepwise regression analysis, we developed linear predictive models for each city across the observed years, culminating in a total of 752 models for the 107 cities studied (Fig. 5a). These models exhibited an average explanatory power ranging from 82% to 87% for the eight levels of SWB, with the model significance consistently below 0.05 (Fig. 5b). Our comprehensive analysis reveals a complex interplay between influencing determinants of SWB. By assessing the frequency of significance testing of these factors within the regression models, we established a hierarchy of importance among the indicators, in descending order: NDVI, SE, AT, CR, RD, GN, GDP, PD, GY, PS, POP and OP (Fig. 5c). This hierarchy underscores the predominant influence of ecological factors over socioeconomic factors in determining SWB.

## DISCUSSION AND CONCLUSIONS

This multidimensional study significantly enhances our understanding of the influence of urbanization-induced socioecological changes on SWB. We found that the trajectory of SWB does not consistently align with economic growth and urban expansion, highlighting the complex nature of this relationship. Interestingly, our results reveal a divergence from the broader literature, which often assumes a linear relationship between economic indicators and life satisfaction or emotional well-being [36]. Our findings offer valuable insights for urban planners and policymakers who aim to create environments that improve the quality of life for residents.

Previous studies have shown a reduction in the SWB disparity between different income groups [33] and between urban and rural areas in China [34]. Our findings align with these global trends, suggesting a potential ‘levelling effect’ of urbanization on well-being disparities. Notably, our findings reveal a widening disparity in SWB along the urbanization gradient—from U1 (earlier urbanized city center areas) to U30 (newer districts)—as illustrated in Fig. 2b. However, new urbanization has led to an increase in SWB and more balanced planning, thereby reducing the SWB gap among residents. This highlights the importance of human-centered urban planning and policy in shaping the well-being landscape. China's One Health strategies need to consider the transformation of urban and rural areas and its impact on SWB [37,38]. The negative non-universal association between AT and SWB among urban SWB groups highlights complex contextual dynamics and reveals important psychophysiological mechanisms underlying thermal comfort preferences in urbanized environments (Fig. 3). Research in environmental psychology demonstrates that thermal discomfort significantly impairs cognitive function and emotional regulation, with these effects particularly pronounced among individuals with higher baseline well-being who may have heightened sensitivity to environmental deviations from optimal conditions [40]. Another intriguing finding is that socioeconomic indicators have increased high SWB and reduced low SWB, thereby widening the SWB disparity. Conversely, urban greening has enhanced low SWB, thus reducing SWB disparity. China's urbanization over the past 30 years has shown that the impact of urban greening on SWB outweighs the socioeconomic impact, leading to a reduction in SWB disparity. This suggests that enhancing ecological measures is crucial as we proceed with the revitalization of old urban areas. These findings provide valuable lessons for other developing countries aiming to balance urbanization and SWB.

A recent study examines spatial inequality across Chinese cities, revealing how socioeconomic segregation creates disparate access patterns to critical well-being resources [25]. Physical urban configurations increasingly separate population groups along socioeconomic lines, resulting in stratified accessibility to essential amenities including healthcare facilities, educational institutions, cultural spaces and high-quality green areas. These structured disparities in environmental resources systematically shape residents’ daily lived experiences and create persistent well-being disadvantages for marginalized urban populations. Our study further highlights the crucial role of urban green spaces in enhancing SWB. This aligns with an expanding body of research that emphasizes the mental and physical health benefits associated with connectivity to nature [41–43]. These benefits include promoting physical health, alleviating lifestyle anxieties and fostering social interaction. Our study reveals an improvement in urban green landscapes across China during the last 30 years. While urban green spaces can provide a variety of ecosystem services, it is important to note that they significantly differ from natural ecosystems and can notably influence urban biodiversity [44]. Additionally, there are trade-offs among different ecosystem services, as overemphasizing one function may potentially limit others [45,46]. The varying impact of urban greening initiatives across different demographic groups underscores the need for a comprehensive and inclusive approach to urban planning. This approach should prioritize equitable access to green spaces, ensuring their benefits are fairly distributed for all population segments.

The relationship between urban expansion rate and SWB is examined across 107 of China's cities (Fig. S11), revealing a complex, non-linear relationship between urbanization expansion rates and SWB. Cities with moderate urbanization rates display considerable variability in SWB outcomes. It suggests a potential threshold effect where extremely high urbanization rates are associated with relatively stable but not necessarily higher SWB scores. These regional variations provide important context for understanding how urbanization processes interact with local socioeconomic conditions and cultural factors to shape well-being outcomes. The clustering pattern highlights differentiated urbanization strategies based on regional SWB patterns. At the national level, rapid urbanization does not automatically translate to higher SWB. Policymakers should consider optimal urbanization paces that allow for social and infrastructure adaptation rather than pursuing maximum expansion rates. The convergence of SWB scores at higher urbanization rates suggests diminishing well-being returns from continued rapid expansion. Policy priorities might better focus on qualitative improvements to existing urban environments rather than continued expansion. This evidence suggests that optimal urbanization policies should prioritize balanced development approaches that consider SWB outcomes alongside physical expansion metrics, with particular attention to the quality of urban growth rather than simply its pace or scale.

Our urbanization gradient framework (U1–U30) inherently captures the spatial signatures of different expansion patterns, reflecting varying intensities of urban development that result from different expansion trajectories. Different urban expansion patterns—including compact development, sprawling growth, leapfrog development and edge expansion—create distinct socioecological contexts that can differentially impact residents’ well-being [47,48]. Compact urban growth typically promotes walkability, social cohesion and access to services, potentially enhancing SWB through improved social connectivity and reduced commuting stress. Conversely, sprawling development patterns may negatively affect SWB through increased social isolation, longer commutes and reduced access to urban amenities, while potentially providing benefits through larger living spaces and closer contact with nature. Our findings suggest that the relationship between urban environmental factors and resident well-being is not uniform across all urban contexts but varies systematically with the underlying spatial structure created by different expansion patterns. This has important implications for place-based urban policies aimed at enhancing resident well-being.

The nuanced insights from our analysis into the relationships between urbanization, green spaces and SWB have profound implications for the advancement of SDGs. In light of our findings, we advocate for a paradigm shift in urban development policies, moving beyond conventional economic metrics towards a more holistic evaluation framework that incorporates SWB indicators. The adoption of alternative indices such as gross happiness production (GHP) and the happy planet index (HPI) reflects a growing recognition of the limitations of GDP as the sole measure of social progress. It underscores the need for a more comprehensive approach to assessing human well-being and sustainability [49]. Our models account for 82%–87% of the variance in SWB, but other factors, such as governance quality and social cohesion, may further improve explanatory power. Due to data constraints, these variables were excluded from this analysis. We acknowledge their potential significance and plan to include them in future research as data availability improves. Besides, the preponderance of younger users on Weibo may skew our SWB measurements toward the perspectives and experiences of the relatively young group (Fig. S9). Younger individuals often exhibit distinct psychological and social behaviors compared to older populations, such as higher sensitivity to social media-driven social comparisons or different expectations of urban environments. This demographic bias suggests that our findings may overrepresent the SWB priorities of younger urban residents, potentially underestimating the importance of socio-institutional factors like public-service accessibility for broader populations.

In conclusion, our study contributes to the evolving narrative on urbanization and SWB, highlighting the complexity of their interplay and the critical need for integrated, well-being-centric urban development strategies. As we move forward, it is imperative that future research expands the scope of inquiry to include cross-cultural analyses and longitudinal studies that can offer deeper insights into the global dynamics of urbanization and well-being. Such efforts will enhance our understanding of SWB determinants across different urban contexts. They will also guide the development of policies and strategies to address urbanization challenges while promoting sustainable and inclusive well-being.

## METHODOLOGY

To explore SWB changes over time, we utilize a mixed methodology (Fig. S14). Initially, we measure urbanization intensity and SWB with urban big data, incorporating NTL and social media posts, offering a thorough perspective on SWB trends during urbanization. Subsequently, we examine the spatial-temporal SWB patterns through regional statistical methods. Lastly, we evaluate residents’ SWB using the SE and socioeconomic factors like the NDVI, AT, RD, POP and PS. To ensure data reliability, we chose 107 Chinese cities meeting specific criteria for our case study: cities with over 10 urban expansion boundaries in the previous 30 years, at least 50 posts in each expansion area, and a minimum of 2000 street view images. This approach guarantees our analysis is grounded in extensive datasets and robust analytic methodologies.

### Urbanization gradient delineation

We delineated urban extents using the natural city framework, which defines settlements objectively based on patterns of human activity rather than administrative boundaries. These natural cities emerge organically from geographic information analysis and are systematically identified through the head/tail division rule methodology [3]. This approach captures the functional urban footprint as revealed through actual spatial patterns of human presence and interaction. The head/tail division rule leverages the inherently unbalanced statistical properties of urban systems, considering an unbalanced distribution for a certain variable that follows a heavy-tailed distribution.

Using the head/tail break method, we apply NTL data to delineate and analyze urban expansion boundaries for each city across from 1992 to 2021 [50]. The NTL data combine essential datasets from two primary sources: the Defense Meteorological Satellite Program (DMSP) and the Visible Infrared Imaging Radiometer Suite (VIIRS). Initially, we calculate the mean value of an original NTL image as the initial threshold. This separates the pixels with values above the mean as the head, while those below the mean are considered the tail. This process is repeated until the number of pixels above the mean equals those below it. The mean value from the final iteration is then used as the threshold to differentiate the head and tail. Consequently, this allows us to define the core urban areas in China using these homogeneous pixels derived from the NTL data [51]. We then use the urban land expansion rate as a quantitative measure of the pace at which urban areas have expanded over a specified period. In this study, we define the urban land expansion rate (r) as follows:


(1) \begin{eqnarray*}
r = \frac{1}{{N - 1}}\mathop \sum \limits_i^{N - 1} \frac{{{A}_{i + 1} - {A}_i}}{{{A}_i}} \times 100\%,
\end{eqnarray*}

where *N* represents the number of years considered for analysis, specifically *N* = 30 in our study, and *A*_i_ symbolizes the land area occupied by a city in the *i*th year.

### SWB quantification

Utilizing crawler technology, we obtain 6.39 million posts across China from the Weibo platform (Sina microblog) [52]. These posts, contributed by 3.83 million individuals from September 2020 to April 2021, encompass crucial information such as the time of publication, geolocation (latitude and longitude) and text content. Weibo ranks as one of the top social networking platforms, boasting 511 million monthly active users in 2020 [53,54]. According to the Weibo 2020 user development report, the total proportion of post-90s and post-00s groups accounts for nearly 80%. Generation *Z* is the primary user group of Weibo [55]. Before analyzing the SWB of these social media posts, we take two crucial preprocessing steps to ensure data validity. These steps include eliminating emojis and place names from the posts, as well as filtering out posts with fewer than five Chinese characters. Based on the natural boundaries of 107 cities, we build a dataset composed of 5.3 million social media posts. The average post count in these cities is 49 700, ranging from 2 000 to 710 000. The average count of Chinese characters in the social media posts dataset is 20. The BERT-wwm Chinese pre-training model is an improved method based on the pre-trained language model BERT. We use the BERT-wwm model based on whole word masking to transform sentences into word vectors [56]. In the BERT-wwm model, the complete forms of low-frequency words are retained more effectively by adopting a more rational WordPiece sampling strategy, instead of breaking them into finer-grained fragments. This helps the model better learn the semantic information of low-frequency words, thus improving its performance. Additionally, BERT-wwm better adapts to the characteristics of Chinese text by improving the training sample generation strategy. We then train a sentiment scoring model for social media posts using a logistic regression classifier on the first 100 principal component analysis dimensions of BERT-wwm [57]. Based on this model, we calculate the SWB for all the Weibo media posts. We calculate the 10%, 20%, 30%, 40%, 50%, 60%, 70% and 80% quantiles of SWB values within each expansion area. These quantiles serve as a metric to represent the levels of SWB among different segments of the population:


(2) \begin{eqnarray*}
{P}_i = {\mathrm{\ }}\mathit{percentile}\left( {A,i} \right){\mathrm{\ }}\left( {i = 0.1,0.2,\ldots,0.8} \right),\nonumber\\
\end{eqnarray*}

where *P_i_* denotes the *i*th percentile of the SWB values within the urban land expansion area of a city, while *A* represents the set of SWB values of all social media posts. Both *P_i_* and *A* fall within the range of [0,1].

The BERT-wwm model underwent comprehensive fine-tuning using a strategically partitioned dataset of 10 000 manually annotated Weibo posts. We implemented a rigorous data-splitting strategy (64% training, 16% validation, 20% held-out test) to ensure robust model evaluation. Hyperparameter optimization identified optimal training configurations: learning rate of 7e-3 with linear warmup over 3 epochs, hidden representation dimension of 768, training across 20 epochs with early stopping (patience = 3), batch size of 128 and weight decay rate of 0.01 to prevent overfitting. The model achieved strong performance metrics on the held-out test set with 80% classification accuracy across all sentiment categories and an area under the receiver operating characteristic curve (AUC-ROC) of 85%, indicating excellent discriminative capacity. Cross-validation confirmed performance stability across different data subsets.

Our sentiment analysis framework leverages BERT's architectural advantages over traditional bidirectional long short-term memory (BiLSTM) approaches through several key mechanisms. First, BERT's bidirectional transformer architecture with dynamic contextual word embeddings and multi-head self-attention captures the subtle emotional nuances and complex contextual semantics characteristic of urban discourse on Weibo. Second, BERT's extensive pre-training on diverse Chinese corpora (over 5.4 billion tokens) enables robust understanding of non-standard expressions, regional colloquialisms and implicit cultural knowledge frequently embedded in Weibo discussions about urban environments. Third, BERT's sophisticated word segmentation and word tokenization strategies effectively handle the specialized terminology, neologisms and creative language constructions prevalent in Chinese social media content, particularly when discussing emerging urban phenomena. These capabilities proved crucial for accurately classifying the complex sentiment patterns expressed in urbanization-related discussions.

### SE quantification

Based on 2.92 million street view images, we quantify SE factors. Initially, we utilize the pre-trained DeepLabv3 model for street view image recognition (Fig. S15). The pre-trained DeepLabv3 model employs deep convolutional neural networks, enhanced by atrous convolution and an atrous spatial pyramid pooling (ASPP) module [58], to effectively capture multi-scale contextual information. This setup addresses segmentation challenges presented by objects of varying sizes in street scenes. Moreover, its encoder–decoder structure preserves spatial information, making it highly suitable for processing complex scenes and detail-rich datasets. This structure enables the model to excel in recognizing details within street scenes. Additionally, the pre-trained DeepLabv3 model, having been trained on a substantial volume of data, possesses strong generalization capabilities to adapt to various street scenes, providing an accurate and reliable technical solution for street scene recognition [59].

This study identifies 19 typical scene elements from street view images, including roads, sidewalks, buildings, walls, fences, traffic poles, traffic lights, traffic signs, vegetation, ground, sky, pedestrians, cyclists, cars, trucks, buses, trains, motorcycles and bicycles. We accurately identify pixel objects in the street view images with an accuracy of 80.2% [60]. Secondly, we design four indicators: GN, GY, OP and CR. These indicators represent four key aspects of urban areas: the ecological environment, urban development, urban morphology and anthropogenic activities. Lastly, we integrate these four indicators to construct the urban SE (Fig. S7). The following formulae are used to calculate the SE:


(3) \begin{eqnarray*}
\rm SE = \left( {\rm GN \times \rm {OP}} \right)/\left( {\rm {GY} \times \rm {CR}} \right),\end{eqnarray*}

(4) \begin{eqnarray*}\rm GN = \mathop \sum \limits_i^N \left( {\mathit{vegetation}_i/su{m}_i} \right),\end{eqnarray*}

(5) \begin{eqnarray*}\rm GY &=& \mathop \sum \limits_i^N [\left( {\mathit{building}_i + wal{l}_i + \mathit{fence}_i + pol{e}_i}\right.\nonumber\\
&&+\,\left. {\mathit{trafficlight}_i + \mathit{trafficsign}_i} \right)/su{m}_i],\end{eqnarray*}

(6) \begin{eqnarray*}\rm OP = \mathop \sum \limits_i^N [\left( {\mathit{terrain}_i + sk{y}_i + roa{d}_i + \mathit{sidewalk}_i} \right)/su{m}_i],\end{eqnarray*}

(7) \begin{eqnarray*}\rm CR &=& \mathop \sum \limits_i^N [\left( {\mathit{person}_i + \mathit{rider}_i + ca{r}_i + \mathit{truck}_i + bu{s}_i }\right.\nonumber\\
&&+\,\left. {\mathit{train}_i + \mathit{motorcycle}_i + \mathit{bicycle}_i} \right)/su{m}_i],\end{eqnarray*}

where GN, GY, OP and CR are street ecological descriptors. The variables road, sidewalk, building, wall, fence, pole, traffic light, traffic sign, vegetation, terrain, sky, person, rider, car, truck, bus, train, motorcycle and bicycle represent the pixel counts corresponding to the landscape element, and *N* represents the number of images obtained at a collection point. In this study, we used four images from angles of 0°, 90°, 180° and 270°, namely, *N* = 4. *i* represents the image order number.

### Other social and ecological factors

The selection of socioecological indicators was informed by both theoretical foundations in urbanization-wellbeing research and practical considerations related to data availability and consistency across urbanization gradients. The NDVI was selected as a representative indicator, supported by extensive empirical evidence linking greenspace exposure to various dimensions of SWB [61–63]. Higher vegetation density has been consistently associated with reduced psychological distress, improved cognitive restoration and enhanced life satisfaction [23,64,65]. Normalized difference vegetation index values are computed from the worldwide Sentinel-2 dataset, which provides a 10 m spatial resolution spanning September 2020 to April 2021. We aggregate these values using the maximum value composite method to eliminate interference from clouds and the atmosphere. Urbanization and population densification create distinct urban heat islands and microclimate variations, with empirical evidence demonstrating that ambient AT significantly influences psychological comfort, thermal stress perception and overall SWB across seasonally variable urban environments [66–68]. Therefore, we incorporate AT as a key climate variable in our analytical framework, examining both its systematic variation across the urbanization gradient and its contribution to SWB outcomes. The average AT at a 2-m height (ERA5-Land) for the same period is chosen as a crucial indicator of overall climatic conditions. This data's timeframe aligns with the social media posts’ acquisition time.

Road network density was included as a multidimensional indicator reflecting both the level of physical infrastructure and accessibility to resources [24]. It influences individuals’ activity spaces and opportunities for social interaction, key determinants of SWB. Public-service accessibility was incorporated as a socio-institutional indicator, which emphasizes that well-being is contingent upon individuals’ access to essential services and opportunities [69]. Road networks and PS data sourced from OpenStreetMap (OSM) encompass service-oriented points of interest like public toilets, gas stations, government agencies, repair shops, shopping centers, tourist attractions and dining establishments. The network data can be accessed at https://download.geofabrik.de/. Finally, POP was selected as a core demographic indicator, given its impact on social interaction patterns, perceptions of privacy and stress levels [70]. The WorldPop global gridded population datasets for 2020, with a 100 m spatial resolution, are merged within each urban expansion zone. Furthermore, we incorporated 1 km gridded GDP data as a socioeconomic driver [71]. We used GDP data from 2019, which most closely corresponds to the SWB data collection period (2020 and 2021), across the selected 107 cities in China.

### Statistical analysis

This study employs a multivariate stepwise regression framework to systematically examine how socioecological factors influence SWB across China's urban–rural continuum. Considering the differences in urbanization characteristics over a nationwide scale, the core processes of stepwise regression involve introducing variables one at a time and removing those deemed irrelevant to ensure that each variable in the regression model holds significant meaning (Fig. S8). Upon introducing a new variable, we first check whether the variable causes a significant change in the model via an *F*-test. If it does, a *T*-test is conducted. If a previously introduced variable becomes insignificant due to the addition of subsequent variables, it is removed. This process ensures that only significant variables are included in the regression equation before introducing each new variable. The stepwise regression is conducted based on eight SWB levels (from the 10th to the 80th percentiles) for each year of 107 cities (Equation 8). The importance of each indicator is quantified based on the frequency it is retained in these models.


(8) \begin{eqnarray*}P &=& {\beta }_1 \times \rm SE + {\beta }_2 \times \mathrm{NDVI} + {\beta }_3 \times \rm AT + {\beta }_4\nonumber\\
&&\times\, \rm RD + {\beta }_5 \times \rm PD + {\beta }_6 \times \rm PS + {\beta }_0,
\end{eqnarray*}

where *P_i_* denotes the *i*th percentile of the SWB values within the urban land expansion area of a city, *β*_1_, *β*_2_, *β*_3_… *β*_6_ represent the regression coefficients, and *β*_0_ represents the regression constant.

Meanwhile, we adopted GAMs, Geodetector analysis and machine learning algorithms (XGBoost) to further examine the beyond-linear relationships between socioecological factors and SWB.

## Supplementary Material

nwaf362_Supplemental_File

## Data Availability

Data will be made available on request.
