# Supplementary material for: Declining subjective well-being disparities concurrent with urbanization in China
Source: Natl Sci Rev. 2025 Aug 29;12(10):nwaf362. doi: 10.1093/nsr/nwaf362 (PMC12485612; doi:10.1093/nsr/nwaf362)
Supplement: nwaf362_Supplemental_File [file nwaf362_supplemental_file.docx]

# Supplementary Tables

Supplementary Table 1 offers additional details related to Figure 1 in the Results section. This table lists the 107 cities studied, along with the geometric centers of their administrative boundaries. It also provides each city's expansion rate, representing the average growth rate of the urban area observed over the past 30 years. Supplementary Table 2 supplements Figure 2 in the Results section with data including the count of social media posts used to calculate subjective well-being (SWB) for each city. It also includes SWB statistics such as mean, median, variance, and eight percentile values (P1-P8, from the 10^th^ to the 80^th^ percentiles).

## Supplementary Table 1. Urban expansion rates in 107 Chinese cities from 1992 to 2021.

| **City** | **Longitude (°E)** | **Latitude (°N)** | **Expansion rate (km^2^ yr^-1^)** |
| --- | --- | --- | --- |
| Beijing | 116.41 | 40.19 | 147.33 |
| Tianjin | 117.33 | 39.29 | 153.96 |
| Shijiazhuang | 114.44 | 38.13 | 50.68 |
| Tangshan | 118.34 | 39.72 | 84.46 |
| Handan | 114.54 | 36.55 | 27.5 |
| Baoding | 115.17 | 39.02 | 55.61 |
| Chengde | 117.55 | 41.35 | 20.9 |
| Langfang | 116.63 | 39.26 | 59.47 |
| Taiyuan | 112.32 | 37.96 | 28.6 |
| Jinzhong | 112.96 | 37.33 | 24.7 |
| Yuncheng | 111.06 | 35.19 | 20.1 |
| Hohhot | 111.5 | 40.59 | 33.63 |
| Shenyang | 123.14 | 42.09 | 65.54 |
| Dalian | 122.21 | 39.59 | 36.14 |
| Dandong | 124.4 | 40.54 | 9.01 |
| Yingkou | 122.46 | 40.39 | 27.84 |
| Changchun | 125.77 | 44.38 | 65.25 |
| Harbin | 127.96 | 45.64 | 47.18 |
| Daqing | 124.7 | 46.35 | 35.61 |
| Shanghai | 121.47 | 31.24 | 131.76 |
| Nanjing | 118.84 | 31.93 | 100.61 |
| Wuxi | 120.08 | 31.53 | 125.74 |
| Changzhou | 119.64 | 31.63 | 85.76 |
| Suzhou | 120.65 | 31.38 | 257.83 |
| Nantong | 121.02 | 32.19 | 84.48 |
| Yangzhou | 119.47 | 32.74 | 55.62 |
| Zhenjiang | 119.45 | 32.02 | 67.82 |
| Taizhou | 120.06 | 32.57 | 66.43 |
| Hangzhou | 119.47 | 29.9 | 117.17 |
| Ningbo | 121.46 | 29.74 | 132.04 |
| Wenzhou | 120.44 | 27.9 | 80.33 |
| Jiaxing | 120.8 | 30.62 | 108.99 |
| Huzhou | 119.87 | 30.74 | 59.38 |
| Shaoxing | 120.64 | 29.74 | 71.79 |
| Jinhua | 119.95 | 29.12 | 93.79 |
| Quzhou | 118.67 | 28.93 | 21.26 |
| Taizhou | 121.11 | 28.77 | 71.84 |
| Lishui | 119.51 | 28.2 | 26.34 |
| Hefei | 117.36 | 31.76 | 71.9 |
| Wuhu | 118.13 | 31.16 | 43.17 |
| Anqing | 116.49 | 30.58 | 23.21 |
| Fuzhou | 119.18 | 26.05 | 76.67 |
| Xiamen | 118.12 | 24.68 | 28.97 |
| Putian | 118.89 | 25.45 | 38.75 |
| Quanzhou | 118.26 | 25.2 | 107.31 |
| Nanping | 118.14 | 27.34 | 20.91 |
| Nanchang | 116.02 | 28.65 | 42.51 |
| Jiujiang | 115.45 | 29.32 | 29.25 |
| Jinan | 117.21 | 36.64 | 69.53 |
| Qingdao | 120.14 | 36.45 | 68.26 |
| Zibo | 118.05 | 36.61 | 40.33 |
| Yantai | 120.8 | 37.24 | 47.11 |
| Weifang | 119.07 | 36.55 | 81.29 |
| Jining | 116.73 | 35.37 | 50.25 |
| Taian | 117.03 | 36 | 19.85 |
| Weihai | 121.98 | 37.12 | 26.43 |
| Rizhao | 119.14 | 35.58 | 19.06 |
| Linyi | 118.28 | 35.31 | 68.84 |
| Zhengzhou | 113.47 | 34.63 | 98.31 |
| Luoyang | 112.03 | 34.29 | 44.78 |
| Xinxiang | 114.09 | 35.26 | 36.53 |
| Nanyang | 112.28 | 33.04 | 28.15 |
| Shangqiu | 115.7 | 34.29 | 31.75 |
| Zhoukou | 114.88 | 33.72 | 28.29 |
| Wuhan | 114.34 | 30.62 | 100.95 |
| Yichang | 111.14 | 30.75 | 22.7 |
| Huanggang | 115.34 | 30.72 | 20.5 |
| Xianning | 114.18 | 29.62 | 13.24 |
| Changsha | 113.15 | 28.23 | 69.12 |
| Zhuzhou | 113.52 | 27.12 | 17.4 |
| Hengyang | 112.57 | 26.79 | 18.3 |
| Yueyang | 113.25 | 29.07 | 20.52 |
| Chenzhou | 113.14 | 25.81 | 14.27 |
| Guangzhou | 113.54 | 23.35 | 103.51 |
| Shaoguan | 113.77 | 24.82 | 17.4 |
| Shenzhen | 114.13 | 22.65 | 18.76 |
| Shantou | 116.58 | 23.33 | 29.48 |
| Foshan | 112.94 | 23.01 | 67.03 |
| Jiangmen | 112.67 | 22.28 | 57.36 |
| Zhanjiang | 110.16 | 21.1 | 31.87 |
| Zhaoqing | 112.21 | 23.54 | 33.93 |
| Huizhou | 114.5 | 23.24 | 88.66 |
| Meizhou | 116.08 | 24.2 | 17.82 |
| Shanwei | 115.53 | 23.01 | 25.19 |
| Qingyuan | 112.87 | 24.31 | 41.79 |
| Dongguan | 113.88 | 22.94 | 33.16 |
| Zhongshan | 113.38 | 22.52 | 31.33 |
| Chaozhou | 116.77 | 23.8 | 22.77 |
| Jieyang | 116.12 | 23.34 | 50.05 |
| Yunfu | 111.79 | 22.82 | 12.51 |
| Nanning | 108.46 | 23.06 | 55.64 |
| Liuzhou | 109.37 | 24.95 | 28.82 |
| Guilin | 110.51 | 25.35 | 22.55 |
| Haikou | 110.42 | 19.83 | 18.4 |
| Sanya | 109.42 | 18.39 | 16.42 |
| Chongqing | 107.87 | 30.06 | 191.78 |
| Chengdu | 103.93 | 30.65 | 167.05 |
| Leshan | 103.57 | 29.22 | 27.08 |
| Yibin | 104.64 | 28.57 | 25.66 |
| Guiyang | 106.71 | 26.84 | 53.85 |
| Kunming | 102.87 | 25.39 | 78.21 |
| Lijiang | 100.52 | 26.94 | 9.57 |
| Xi'an | 108.79 | 34.11 | 71.49 |
| Weinan | 109.85 | 34.95 | 28.88 |
| Yan'an | 109.32 | 36.44 | 47.73 |
| Yinchuan | 106.35 | 38.28 | 50.05 |
| Urumqi | 87.79 | 43.75 | 44.78 |

## Supplementary Table 2. Statistics of social media posts for subjective well-being (SWB) across 107 Chinese cities. Eight levels of SWB range from P1 (lowest) to P8 (highest).

| **City** | **Count** | **Mean** | **Median** | **Variance** | **P1** | **P2** | **P3** | **P4** | **P5** | **P6** | **P7** | **P8** |
| --- | --- | --- | --- | --- | --- | --- | --- | --- | --- | --- | --- | --- |
| Beijing | 710779 | 0.82 | 0.89 | 0.03 | 0.56 | 0.72 | 0.8 | 0.85 | 0.89 | 0.92 | 0.94 | 0.95 |
| Tianjin | 94915 | 0.84 | 0.91 | 0.03 | 0.59 | 0.75 | 0.83 | 0.88 | 0.91 | 0.93 | 0.95 | 0.96 |
| Shijiazhuang | 50528 | 0.8 | 0.88 | 0.04 | 0.5 | 0.68 | 0.78 | 0.84 | 0.88 | 0.91 | 0.93 | 0.95 |
| Tangshan | 18207 | 0.82 | 0.9 | 0.04 | 0.54 | 0.71 | 0.8 | 0.86 | 0.9 | 0.92 | 0.94 | 0.96 |
| Handan | 12798 | 0.8 | 0.87 | 0.04 | 0.49 | 0.66 | 0.77 | 0.83 | 0.87 | 0.9 | 0.93 | 0.95 |
| Baoding | 27376 | 0.8 | 0.87 | 0.04 | 0.49 | 0.67 | 0.77 | 0.83 | 0.87 | 0.9 | 0.93 | 0.95 |
| Chengde | 9383 | 0.79 | 0.85 | 0.03 | 0.54 | 0.68 | 0.75 | 0.81 | 0.85 | 0.88 | 0.9 | 0.93 |
| Langfang | 14864 | 0.85 | 0.91 | 0.03 | 0.63 | 0.77 | 0.84 | 0.88 | 0.91 | 0.93 | 0.95 | 0.96 |
| Taiyuan | 41039 | 0.81 | 0.88 | 0.04 | 0.51 | 0.69 | 0.79 | 0.84 | 0.88 | 0.91 | 0.93 | 0.95 |
| Jinzhong | 11440 | 0.78 | 0.84 | 0.04 | 0.5 | 0.65 | 0.74 | 0.79 | 0.84 | 0.87 | 0.9 | 0.93 |
| Yuncheng | 5997 | 0.76 | 0.82 | 0.04 | 0.46 | 0.6 | 0.69 | 0.76 | 0.82 | 0.86 | 0.9 | 0.93 |
| Hohhot | 24127 | 0.69 | 0.75 | 0.05 | 0.35 | 0.51 | 0.62 | 0.69 | 0.75 | 0.8 | 0.85 | 0.88 |
| Shenyang | 97299 | 0.82 | 0.9 | 0.04 | 0.54 | 0.72 | 0.8 | 0.86 | 0.9 | 0.92 | 0.94 | 0.96 |
| Dalian | 78643 | 0.83 | 0.89 | 0.03 | 0.6 | 0.74 | 0.82 | 0.86 | 0.89 | 0.92 | 0.94 | 0.95 |
| Dandong | 8901 | 0.85 | 0.91 | 0.02 | 0.63 | 0.76 | 0.83 | 0.88 | 0.91 | 0.93 | 0.95 | 0.97 |
| Yingkou | 6759 | 0.84 | 0.9 | 0.03 | 0.62 | 0.77 | 0.84 | 0.88 | 0.9 | 0.92 | 0.94 | 0.95 |
| Changchun | 52363 | 0.81 | 0.87 | 0.03 | 0.54 | 0.7 | 0.78 | 0.84 | 0.87 | 0.9 | 0.93 | 0.95 |
| Harbin | 62632 | 0.79 | 0.86 | 0.04 | 0.51 | 0.67 | 0.76 | 0.82 | 0.86 | 0.9 | 0.92 | 0.94 |
| Daqing | 8366 | 0.8 | 0.87 | 0.04 | 0.51 | 0.68 | 0.77 | 0.83 | 0.87 | 0.9 | 0.93 | 0.95 |
| Shanghai | 430522 | 0.88 | 0.93 | 0.02 | 0.71 | 0.83 | 0.88 | 0.91 | 0.93 | 0.95 | 0.96 | 0.97 |
| Nanjing | 187714 | 0.82 | 0.9 | 0.04 | 0.56 | 0.73 | 0.81 | 0.86 | 0.9 | 0.92 | 0.94 | 0.96 |
| Wuxi | 63519 | 0.87 | 0.93 | 0.02 | 0.69 | 0.82 | 0.87 | 0.9 | 0.93 | 0.94 | 0.96 | 0.97 |
| Changzhou | 40628 | 0.83 | 0.9 | 0.04 | 0.56 | 0.74 | 0.82 | 0.87 | 0.9 | 0.92 | 0.94 | 0.96 |
| Suzhou | 134631 | 0.88 | 0.93 | 0.02 | 0.7 | 0.83 | 0.88 | 0.91 | 0.93 | 0.95 | 0.96 | 0.97 |
| Nantong | 19966 | 0.85 | 0.92 | 0.03 | 0.6 | 0.77 | 0.85 | 0.89 | 0.92 | 0.94 | 0.96 | 0.97 |
| Yangzhou | 10851 | 0.84 | 0.91 | 0.03 | 0.57 | 0.74 | 0.83 | 0.88 | 0.91 | 0.93 | 0.95 | 0.97 |
| Zhenjiang | 14709 | 0.86 | 0.92 | 0.03 | 0.64 | 0.78 | 0.85 | 0.89 | 0.92 | 0.94 | 0.95 | 0.97 |
| Taizhou | 4491 | 0.84 | 0.92 | 0.03 | 0.59 | 0.76 | 0.84 | 0.89 | 0.92 | 0.94 | 0.95 | 0.96 |
| Hangzhou | 207384 | 0.87 | 0.93 | 0.03 | 0.68 | 0.81 | 0.87 | 0.9 | 0.93 | 0.94 | 0.96 | 0.97 |
| Ningbo | 69562 | 0.89 | 0.94 | 0.02 | 0.73 | 0.84 | 0.89 | 0.92 | 0.94 | 0.95 | 0.96 | 0.97 |
| Wenzhou | 33356 | 0.86 | 0.92 | 0.03 | 0.64 | 0.78 | 0.85 | 0.89 | 0.92 | 0.94 | 0.95 | 0.97 |
| Jiaxing | 28460 | 0.88 | 0.94 | 0.02 | 0.71 | 0.84 | 0.89 | 0.92 | 0.94 | 0.95 | 0.96 | 0.97 |
| Huzhou | 19408 | 0.88 | 0.93 | 0.02 | 0.73 | 0.84 | 0.89 | 0.91 | 0.93 | 0.95 | 0.96 | 0.97 |
| Shaoxing | 27240 | 0.87 | 0.93 | 0.02 | 0.7 | 0.82 | 0.88 | 0.91 | 0.93 | 0.94 | 0.96 | 0.97 |
| Jinhua | 29726 | 0.83 | 0.9 | 0.03 | 0.59 | 0.74 | 0.82 | 0.86 | 0.9 | 0.92 | 0.94 | 0.96 |
| Quzhou | 4880 | 0.81 | 0.88 | 0.04 | 0.53 | 0.69 | 0.78 | 0.84 | 0.88 | 0.91 | 0.93 | 0.95 |
| Taizhou | 20067 | 0.88 | 0.93 | 0.02 | 0.7 | 0.83 | 0.88 | 0.91 | 0.93 | 0.95 | 0.96 | 0.97 |
| Lishui | 8322 | 0.85 | 0.91 | 0.02 | 0.66 | 0.79 | 0.85 | 0.89 | 0.91 | 0.93 | 0.94 | 0.96 |
| Hefei | 64413 | 0.85 | 0.92 | 0.03 | 0.62 | 0.78 | 0.85 | 0.89 | 0.92 | 0.94 | 0.95 | 0.96 |
| Wuhu | 14230 | 0.81 | 0.88 | 0.03 | 0.55 | 0.71 | 0.79 | 0.84 | 0.88 | 0.9 | 0.92 | 0.94 |
| Anqing | 3769 | 0.78 | 0.86 | 0.05 | 0.45 | 0.62 | 0.73 | 0.81 | 0.86 | 0.9 | 0.93 | 0.95 |
| Fuzhou | 61402 | 0.86 | 0.92 | 0.03 | 0.65 | 0.79 | 0.85 | 0.89 | 0.92 | 0.94 | 0.95 | 0.96 |
| Xiamen | 58930 | 0.85 | 0.91 | 0.03 | 0.65 | 0.78 | 0.84 | 0.88 | 0.91 | 0.93 | 0.94 | 0.96 |
| Putian | 8579 | 0.78 | 0.85 | 0.04 | 0.47 | 0.63 | 0.73 | 0.8 | 0.85 | 0.88 | 0.91 | 0.93 |
| Quanzhou | 34205 | 0.87 | 0.92 | 0.02 | 0.71 | 0.82 | 0.87 | 0.9 | 0.92 | 0.94 | 0.95 | 0.97 |
| Nanping | 6870 | 0.86 | 0.91 | 0.02 | 0.67 | 0.79 | 0.85 | 0.88 | 0.91 | 0.93 | 0.94 | 0.96 |
| Nanchang | 32237 | 0.82 | 0.89 | 0.04 | 0.54 | 0.72 | 0.81 | 0.86 | 0.89 | 0.92 | 0.94 | 0.96 |
| Jiujiang | 9074 | 0.82 | 0.89 | 0.03 | 0.56 | 0.73 | 0.81 | 0.86 | 0.89 | 0.92 | 0.94 | 0.96 |
| Jinan | 94672 | 0.79 | 0.86 | 0.04 | 0.49 | 0.66 | 0.75 | 0.82 | 0.86 | 0.89 | 0.92 | 0.94 |
| Qingdao | 93673 | 0.83 | 0.89 | 0.03 | 0.58 | 0.73 | 0.81 | 0.86 | 0.89 | 0.91 | 0.93 | 0.95 |
| Zibo | 13663 | 0.82 | 0.9 | 0.04 | 0.55 | 0.72 | 0.81 | 0.86 | 0.9 | 0.92 | 0.94 | 0.96 |
| Yantai | 20016 | 0.84 | 0.91 | 0.03 | 0.62 | 0.76 | 0.84 | 0.88 | 0.91 | 0.93 | 0.94 | 0.96 |
| Weifang | 23217 | 0.86 | 0.92 | 0.03 | 0.64 | 0.79 | 0.86 | 0.9 | 0.92 | 0.94 | 0.96 | 0.97 |
| Jining | 15084 | 0.79 | 0.86 | 0.04 | 0.49 | 0.65 | 0.75 | 0.82 | 0.86 | 0.9 | 0.92 | 0.95 |
| Taian | 17996 | 0.8 | 0.87 | 0.03 | 0.54 | 0.69 | 0.77 | 0.83 | 0.87 | 0.9 | 0.92 | 0.94 |
| Weihai | 13097 | 0.85 | 0.9 | 0.03 | 0.66 | 0.78 | 0.84 | 0.87 | 0.9 | 0.92 | 0.94 | 0.96 |
| Rizhao | 9381 | 0.86 | 0.92 | 0.03 | 0.67 | 0.81 | 0.87 | 0.9 | 0.92 | 0.94 | 0.95 | 0.97 |
| Linyi | 16395 | 0.84 | 0.91 | 0.03 | 0.61 | 0.76 | 0.84 | 0.88 | 0.91 | 0.93 | 0.95 | 0.96 |
| Zhengzhou | 111905 | 0.78 | 0.86 | 0.04 | 0.46 | 0.65 | 0.75 | 0.82 | 0.86 | 0.9 | 0.92 | 0.94 |
| Luoyang | 32952 | 0.85 | 0.91 | 0.03 | 0.65 | 0.78 | 0.84 | 0.88 | 0.91 | 0.93 | 0.94 | 0.96 |
| Xinxiang | 12734 | 0.8 | 0.87 | 0.04 | 0.5 | 0.67 | 0.77 | 0.83 | 0.87 | 0.91 | 0.93 | 0.95 |
| Nanyang | 9196 | 0.79 | 0.87 | 0.04 | 0.49 | 0.67 | 0.76 | 0.82 | 0.87 | 0.9 | 0.92 | 0.95 |
| Shangqiu | 5735 | 0.78 | 0.85 | 0.04 | 0.46 | 0.63 | 0.73 | 0.8 | 0.85 | 0.89 | 0.91 | 0.94 |
| Zhoukou | 3750 | 0.81 | 0.88 | 0.03 | 0.55 | 0.71 | 0.79 | 0.85 | 0.88 | 0.91 | 0.93 | 0.95 |
| Wuhan | 184373 | 0.79 | 0.86 | 0.04 | 0.51 | 0.67 | 0.77 | 0.82 | 0.86 | 0.9 | 0.92 | 0.94 |
| Yichang | 17462 | 0.77 | 0.85 | 0.04 | 0.46 | 0.64 | 0.74 | 0.8 | 0.85 | 0.88 | 0.9 | 0.93 |
| Huanggang | 4557 | 0.75 | 0.83 | 0.04 | 0.43 | 0.61 | 0.71 | 0.78 | 0.83 | 0.86 | 0.89 | 0.92 |
| Xianning | 2965 | 0.8 | 0.86 | 0.03 | 0.54 | 0.69 | 0.77 | 0.82 | 0.86 | 0.89 | 0.91 | 0.93 |
| Changsha | 110682 | 0.81 | 0.88 | 0.04 | 0.53 | 0.7 | 0.79 | 0.84 | 0.88 | 0.91 | 0.93 | 0.95 |
| Zhuzhou | 7448 | 0.75 | 0.82 | 0.04 | 0.44 | 0.6 | 0.7 | 0.77 | 0.82 | 0.86 | 0.89 | 0.92 |
| Hengyang | 11569 | 0.8 | 0.87 | 0.04 | 0.49 | 0.66 | 0.76 | 0.83 | 0.87 | 0.9 | 0.93 | 0.95 |
| Yueyang | 5665 | 0.78 | 0.86 | 0.04 | 0.48 | 0.66 | 0.75 | 0.81 | 0.86 | 0.89 | 0.91 | 0.94 |
| Chenzhou | 4771 | 0.8 | 0.87 | 0.04 | 0.51 | 0.67 | 0.77 | 0.83 | 0.87 | 0.9 | 0.92 | 0.94 |
| Guangzhou | 275211 | 0.89 | 0.94 | 0.02 | 0.72 | 0.84 | 0.89 | 0.92 | 0.94 | 0.95 | 0.97 | 0.98 |
| Shaoguan | 8050 | 0.82 | 0.89 | 0.04 | 0.54 | 0.71 | 0.8 | 0.85 | 0.89 | 0.91 | 0.93 | 0.95 |
| Shenzhen | 166322 | 0.86 | 0.92 | 0.03 | 0.66 | 0.8 | 0.86 | 0.89 | 0.92 | 0.94 | 0.95 | 0.97 |
| Shantou | 5757 | 0.89 | 0.93 | 0.01 | 0.77 | 0.85 | 0.89 | 0.92 | 0.93 | 0.95 | 0.96 | 0.97 |
| Foshan | 55265 | 0.91 | 0.95 | 0.02 | 0.78 | 0.88 | 0.91 | 0.94 | 0.95 | 0.96 | 0.97 | 0.98 |
| Jiangmen | 16424 | 0.9 | 0.94 | 0.02 | 0.77 | 0.86 | 0.9 | 0.93 | 0.94 | 0.96 | 0.97 | 0.97 |
| Zhanjiang | 7152 | 0.85 | 0.91 | 0.03 | 0.63 | 0.77 | 0.84 | 0.88 | 0.91 | 0.93 | 0.94 | 0.96 |
| Zhaoqing | 11700 | 0.89 | 0.94 | 0.02 | 0.75 | 0.85 | 0.89 | 0.92 | 0.94 | 0.95 | 0.96 | 0.97 |
| Huizhou | 26012 | 0.9 | 0.94 | 0.02 | 0.76 | 0.86 | 0.9 | 0.93 | 0.94 | 0.96 | 0.97 | 0.98 |
| Meizhou | 4348 | 0.84 | 0.9 | 0.03 | 0.61 | 0.77 | 0.84 | 0.88 | 0.9 | 0.92 | 0.94 | 0.96 |
| Shanwei | 3309 | 0.88 | 0.93 | 0.02 | 0.71 | 0.83 | 0.88 | 0.91 | 0.93 | 0.94 | 0.95 | 0.96 |
| Qingyuan | 11719 | 0.88 | 0.92 | 0.02 | 0.73 | 0.83 | 0.87 | 0.9 | 0.92 | 0.94 | 0.95 | 0.96 |
| Dongguan | 49617 | 0.89 | 0.94 | 0.02 | 0.73 | 0.85 | 0.9 | 0.92 | 0.94 | 0.95 | 0.96 | 0.97 |
| Zhongshan | 20064 | 0.9 | 0.94 | 0.02 | 0.76 | 0.85 | 0.9 | 0.93 | 0.94 | 0.96 | 0.97 | 0.98 |
| Chaozhou | 7578 | 0.91 | 0.95 | 0.01 | 0.81 | 0.88 | 0.91 | 0.93 | 0.95 | 0.96 | 0.97 | 0.98 |
| Jieyang | 8779 | 0.88 | 0.93 | 0.02 | 0.72 | 0.83 | 0.88 | 0.91 | 0.93 | 0.95 | 0.96 | 0.97 |
| Yunfu | 2040 | 0.87 | 0.92 | 0.02 | 0.71 | 0.83 | 0.87 | 0.9 | 0.92 | 0.93 | 0.95 | 0.96 |
| Nanning | 49244 | 0.86 | 0.93 | 0.03 | 0.66 | 0.8 | 0.86 | 0.9 | 0.93 | 0.94 | 0.96 | 0.97 |
| Liuzhou | 14351 | 0.8 | 0.88 | 0.04 | 0.53 | 0.68 | 0.78 | 0.84 | 0.88 | 0.91 | 0.93 | 0.95 |
| Guilin | 27198 | 0.89 | 0.93 | 0.02 | 0.75 | 0.84 | 0.88 | 0.91 | 0.93 | 0.94 | 0.95 | 0.97 |
| Haikou | 20357 | 0.85 | 0.91 | 0.03 | 0.64 | 0.78 | 0.84 | 0.88 | 0.91 | 0.93 | 0.95 | 0.96 |
| Sanya | 30478 | 0.87 | 0.91 | 0.02 | 0.71 | 0.81 | 0.86 | 0.89 | 0.91 | 0.93 | 0.94 | 0.95 |
| Chongqing | 179906 | 0.83 | 0.89 | 0.03 | 0.59 | 0.74 | 0.81 | 0.86 | 0.89 | 0.91 | 0.93 | 0.95 |
| Chengdu | 218826 | 0.82 | 0.89 | 0.03 | 0.56 | 0.73 | 0.81 | 0.86 | 0.89 | 0.92 | 0.94 | 0.95 |
| Leshan | 11500 | 0.86 | 0.9 | 0.02 | 0.71 | 0.81 | 0.85 | 0.88 | 0.9 | 0.92 | 0.93 | 0.95 |
| Yibin | 5041 | 0.87 | 0.93 | 0.02 | 0.7 | 0.82 | 0.88 | 0.91 | 0.93 | 0.94 | 0.96 | 0.97 |
| Guiyang | 32504 | 0.86 | 0.92 | 0.03 | 0.66 | 0.8 | 0.86 | 0.9 | 0.92 | 0.94 | 0.95 | 0.97 |
| Kunming | 72191 | 0.84 | 0.9 | 0.03 | 0.61 | 0.76 | 0.83 | 0.87 | 0.9 | 0.92 | 0.94 | 0.96 |
| Lijiang | 29393 | 0.88 | 0.92 | 0.01 | 0.75 | 0.84 | 0.88 | 0.9 | 0.92 | 0.94 | 0.95 | 0.96 |
| Xi'an | 60569 | 0.81 | 0.87 | 0.04 | 0.53 | 0.69 | 0.78 | 0.83 | 0.87 | 0.9 | 0.93 | 0.95 |
| Weinan | 7483 | 0.79 | 0.86 | 0.03 | 0.53 | 0.68 | 0.77 | 0.82 | 0.86 | 0.89 | 0.91 | 0.94 |
| Yan'an | 4146 | 0.81 | 0.85 | 0.03 | 0.58 | 0.71 | 0.77 | 0.82 | 0.85 | 0.89 | 0.91 | 0.93 |
| Yinchuan | 18265 | 0.81 | 0.88 | 0.04 | 0.54 | 0.71 | 0.79 | 0.85 | 0.88 | 0.91 | 0.93 | 0.95 |
| Urumqi | 29504 | 0.76 | 0.84 | 0.04 | 0.44 | 0.63 | 0.73 | 0.79 | 0.84 | 0.87 | 0.9 | 0.93 |

# Supplementary Figures

For the 107 cities under study, urban boundaries are annually delineated to establish 30 concentric buffer zones, each representing a distinct phase of urban expansion over the three-decade period. Supplementary Figure 1 displays the natural boundaries of 107 cities in China, derived from nighttime light data. Utilizing the DeepLabV3 deep learning algorithm, we identified landscape elements in 2.92 million street view images. From the semantic features of these elements, we developed four descriptors: greenness, greyness, openness, and crowdedness, to depict the ecological pattern of urban streets. Supplementary Figure 2 illustrates the trends of these four descriptors within the expansion areas over different years, which are used to calculate the street ecological index. Supplementary Figure 3 displays the spatial distribution of geotagged posts between September 2020 and April 2021. Supplementary Figure 4a presents the distribution of data at various levels of subjective well-being (SWB) for 107 cities, aiding in understanding the evolving trends of SWB throughout the three decades. Supplementary Figure 4b displays the counts of city development areas over different years alongside their average SWB. Supplementary Figure 5 presents the eight levels of subjective well-being (SWB), from P1 (lowest) to P8 (highest) across 107 Cities. Supplementary Figure 6 illustrates linear correlations between the eight different levels of subjective well-being and the six normalized key influencing factors, by showing that the absolute differences between two fitted lines at the cutoff were all less than 0.05. Supplementary Figure 7 explains the meaning and methodology to calculate the street ecological index using greenness, grayness, openness, and crowdedness.


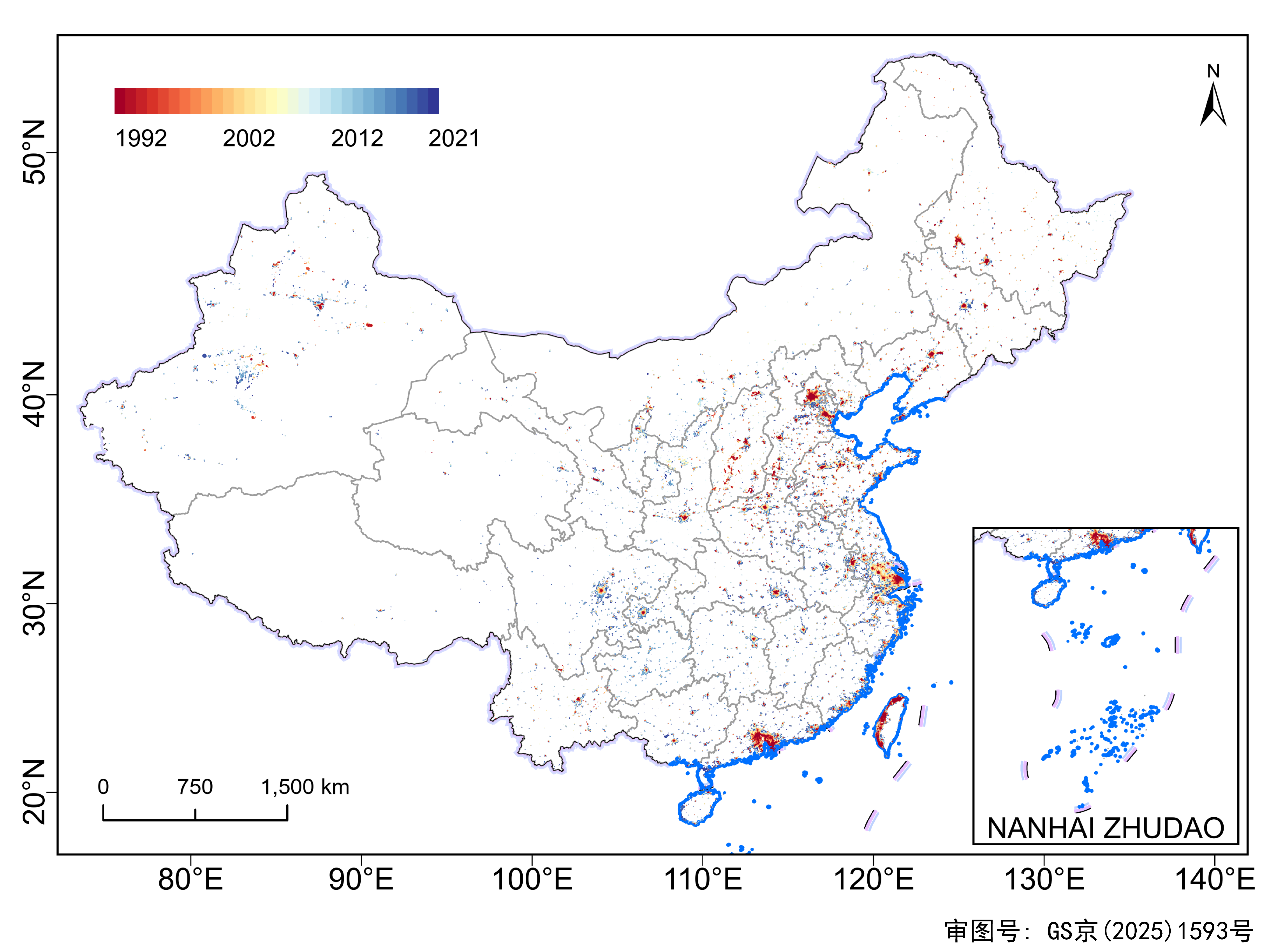


## Supplementary Figure 1. Urban expansion boundaries for Chinese 107 cities from 1992 to 2021.


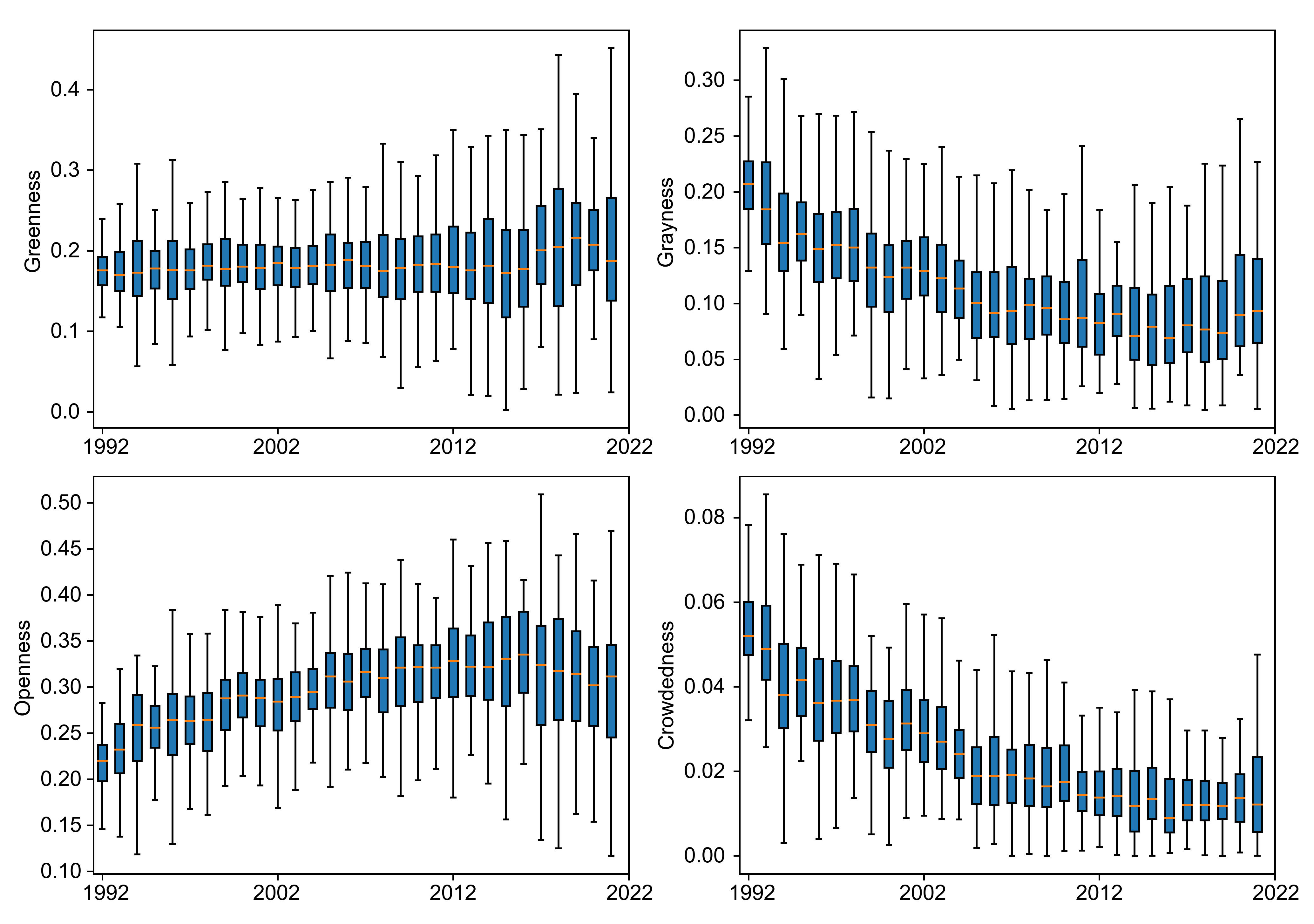


## Supplementary Figure 2. The temporal trends of greenness, grayness, openness, and crowdedness over the last 30 years of urbanization.


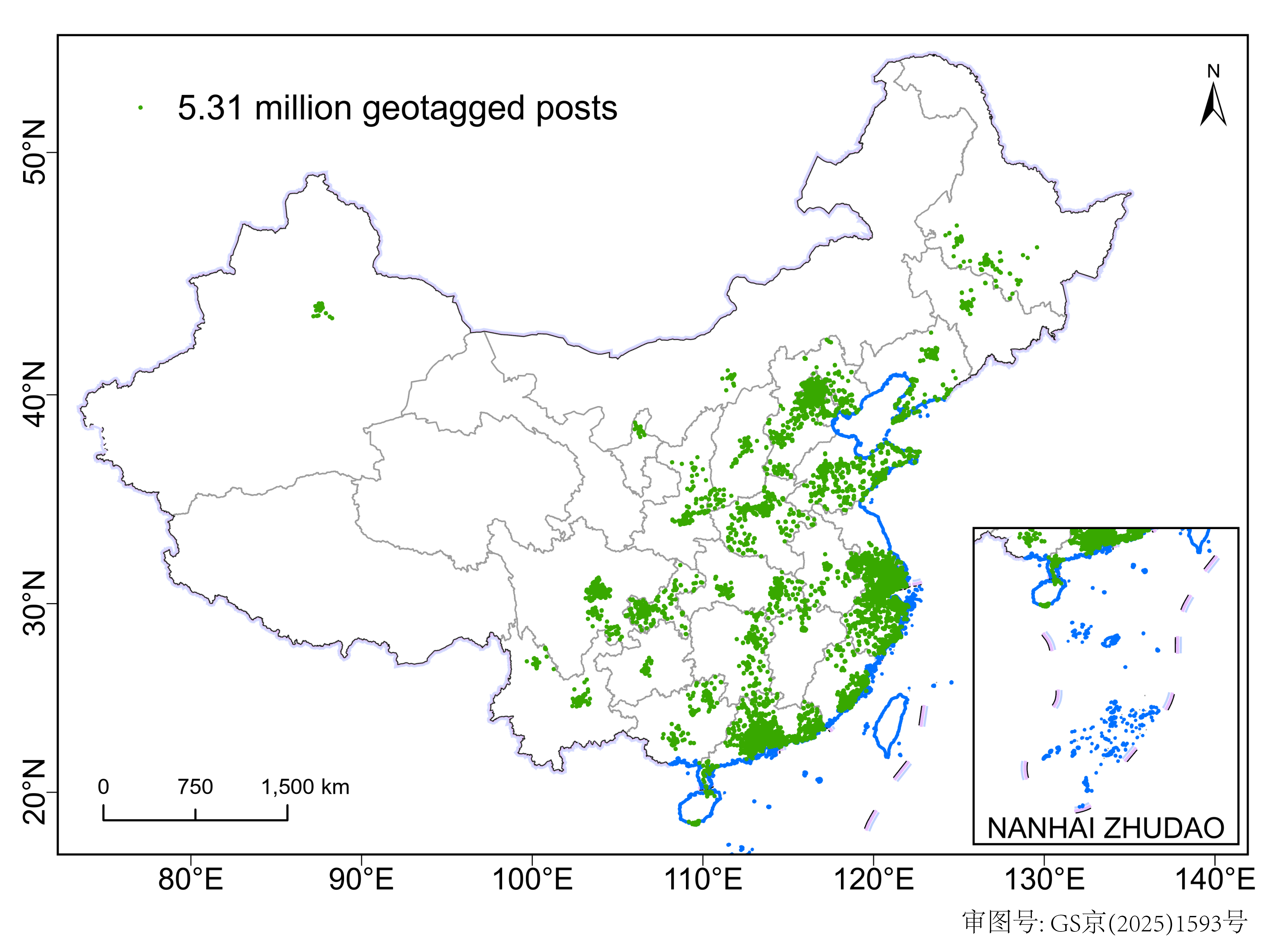


## Supplementary Figure 3. Spatial distribution of 5.31 million geotagged posts generated by 3.83 million users between September 2020 and April 2021 in China.


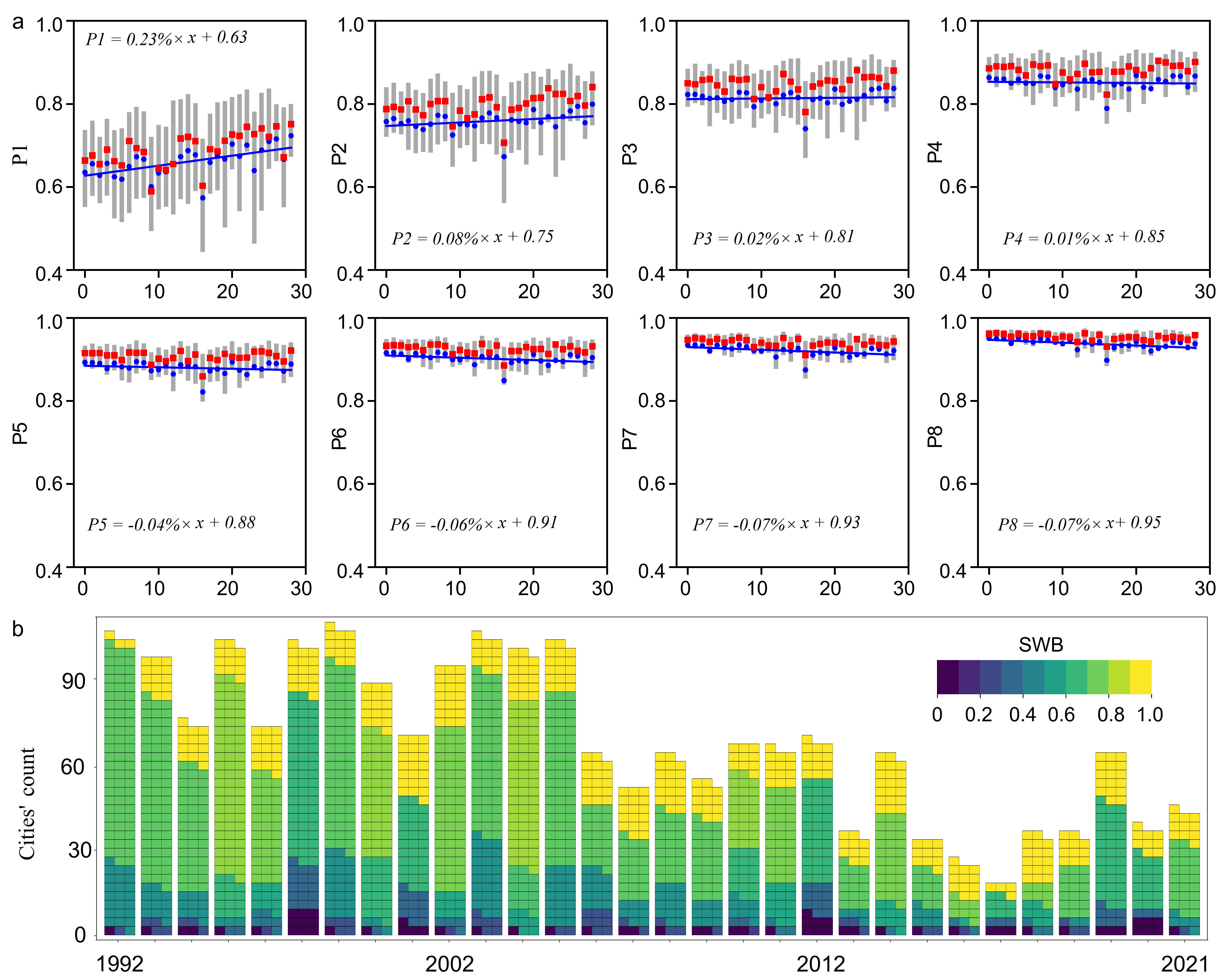


## Supplementary Figure 4. Trends in subjective well-being (SWB) for different SWB groups over 30 years of urbanization. (a) The red and blue dots represent the median and mean values, respectively. The gray lines display the lower 25% and upper 75% quartiles of the SWB levels across 107 Chinese cities in each year. The blue lines display the trend line of the mean values. (b) Dynamics of urban expansion and subjective well-being (SWB) across 30 years of urbanization. The Y-axis denotes the number of cities experiencing expansion in the respective year. Each square represents to an individual city, and the color of the square signifies the average SWB level within that urban expansion area.





## Supplementary Figure 5. Spatial patterns of eight-level subjective well-being (SWB) across 107 Cities. (a-h) Eight levels of SWB range from P1 (lowest) to P8 (highest).


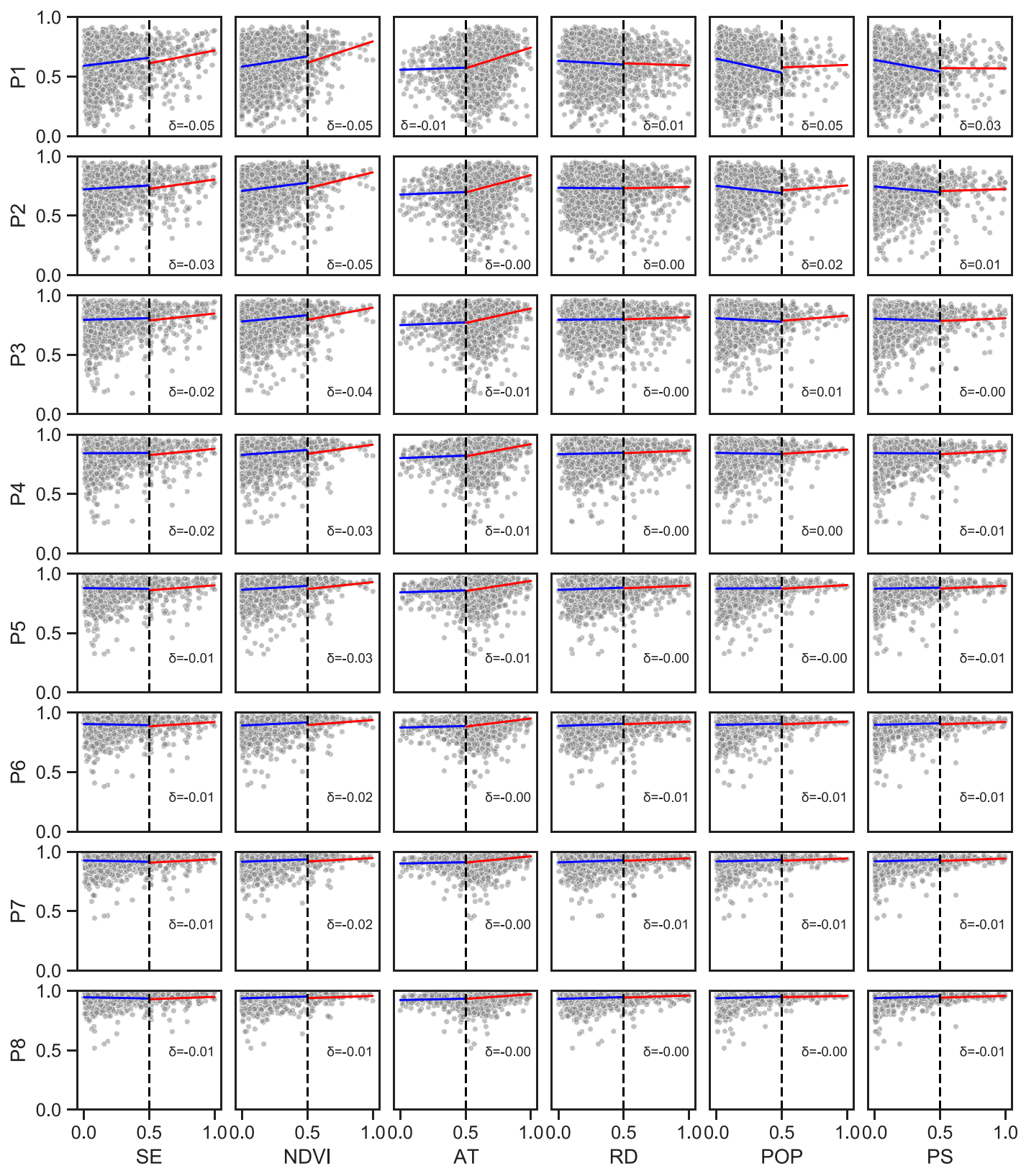


## Supplementary Figure 6. Regression discontinuity design between eight distinct levels of subjective well-being (SWB: P1-P8) and six normalized key influencing factors. These factors include the street ecological index (SE), vegetation index (NDVI), air temperature (AT), road density (RD), population density (POP), and public service facility density (PS). The blue and red lines indicate local linear fits on either side of the cutoff, with δ representing the gap between these fitted lines at the cutoff.


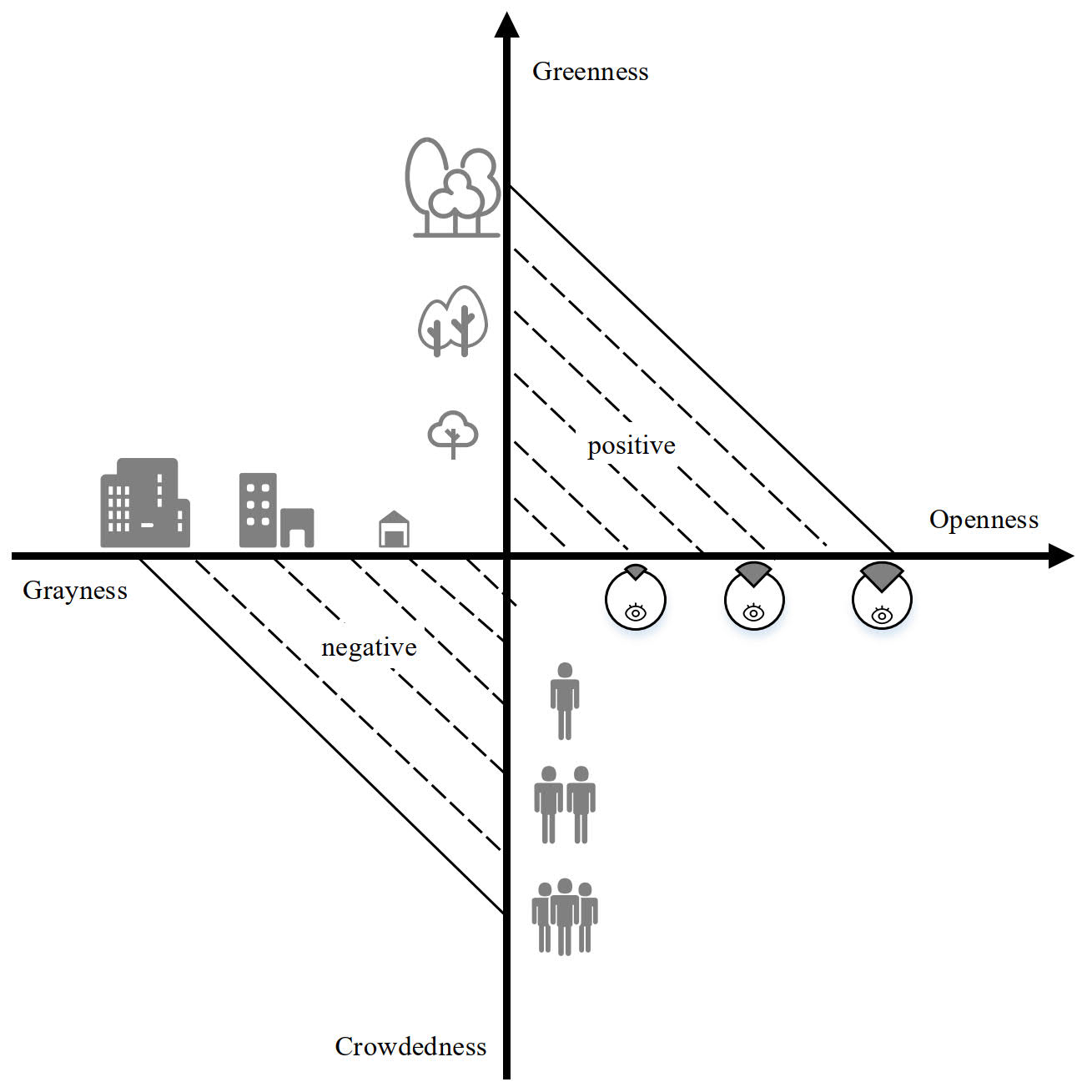

$$\boldsymbol{SE=}\frac{\boldsymbol{Area}_{\boldsymbol{positive}}}{\boldsymbol{Area}_{\boldsymbol{negative}}}\boldsymbol{=}\frac{\boldsymbol{Greenness\times Openness}}{\boldsymbol{Grayness\times Crowdedness}}$$

## Supplementary Figure 7. Schematic diagram of street ecological index (SE) calculation. Greenness, grayness, openness, and crowdedness represent four aspects of urban areas: ecological environment, urban development, urban morphology, and human activities.


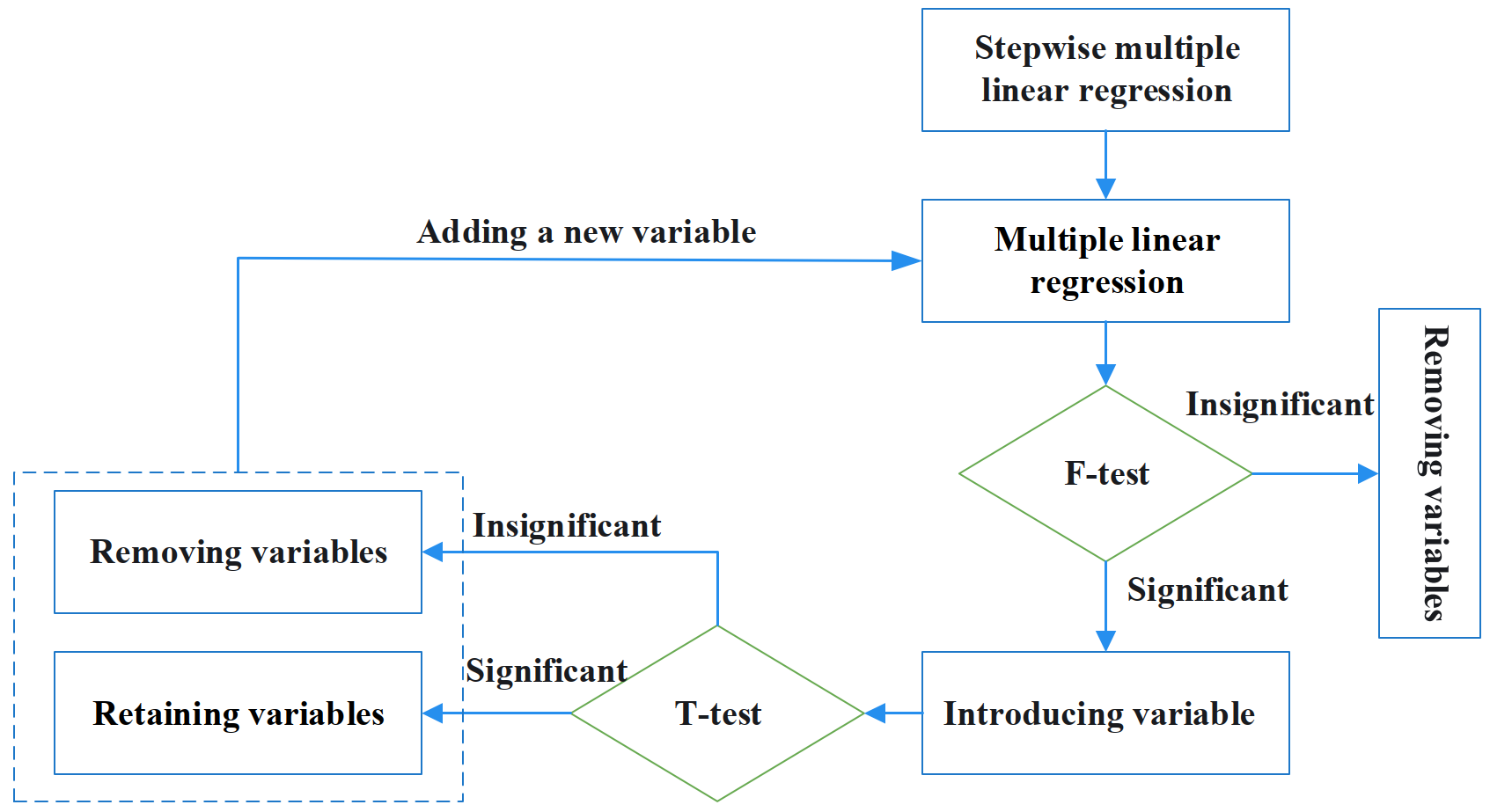


**Supplementary Figure 8.** Stepwise multiple linear regression.

##
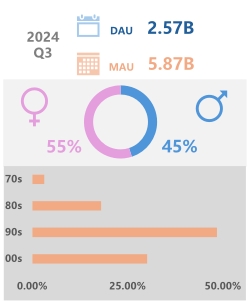


## Supplementary Figure 10. Demographic characteristics in 2024 on the Weibo platform.

##
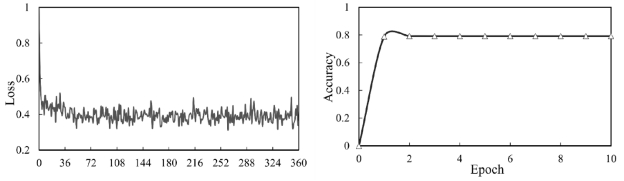


## Supplementary Figure 11 BERT-wwm sentiment model performance and validation

##
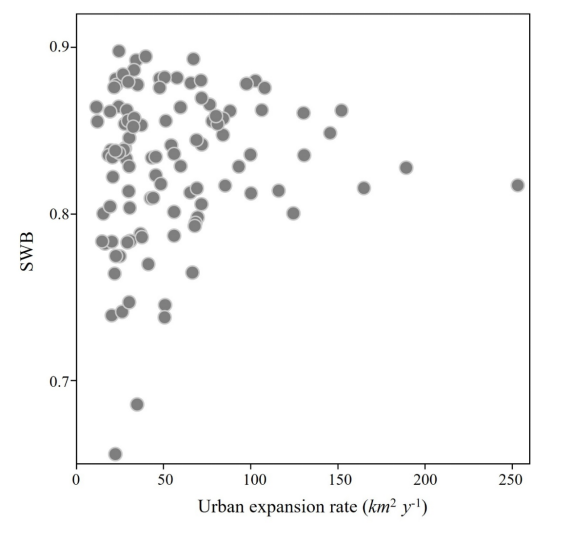


## Supplementary Figure 12 Relationships between urban expansion rate and SWB across 107 cities.

##
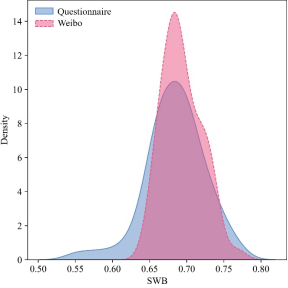

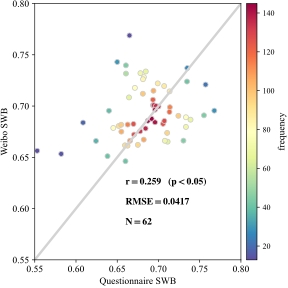


## Supplementary Figure 13 (a) Probability density function of SWB based on the Weibo platform and a nationwide questionnaire-based SWB survey. (b) Comparison in city-wide mean SWB based on the Weibo platform and a nationwide questionnaire-based SWB survey.


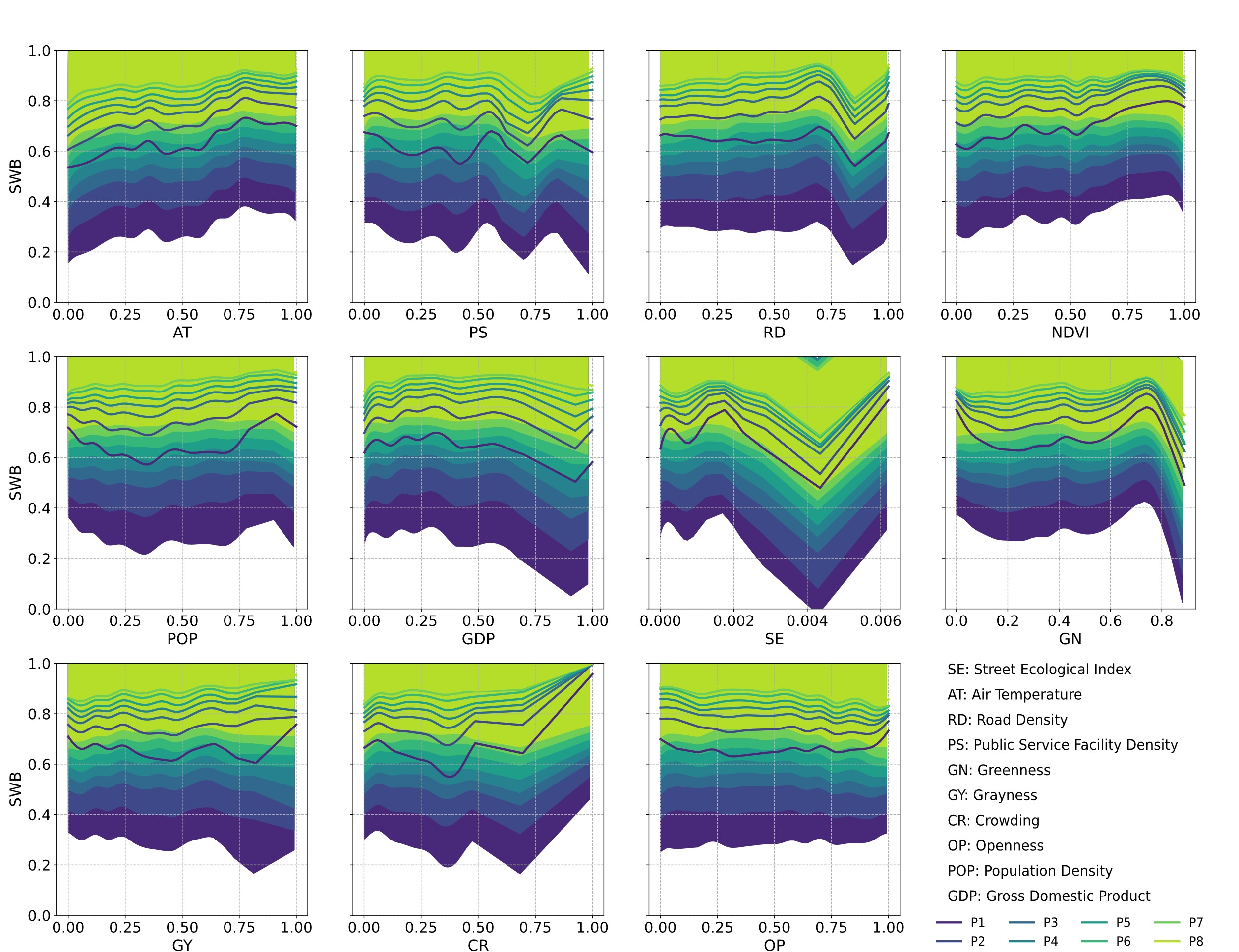


**Supplementary Figure 14**. Relationships between influencing factors and each SWB level derived from the Generalized Additive Models.


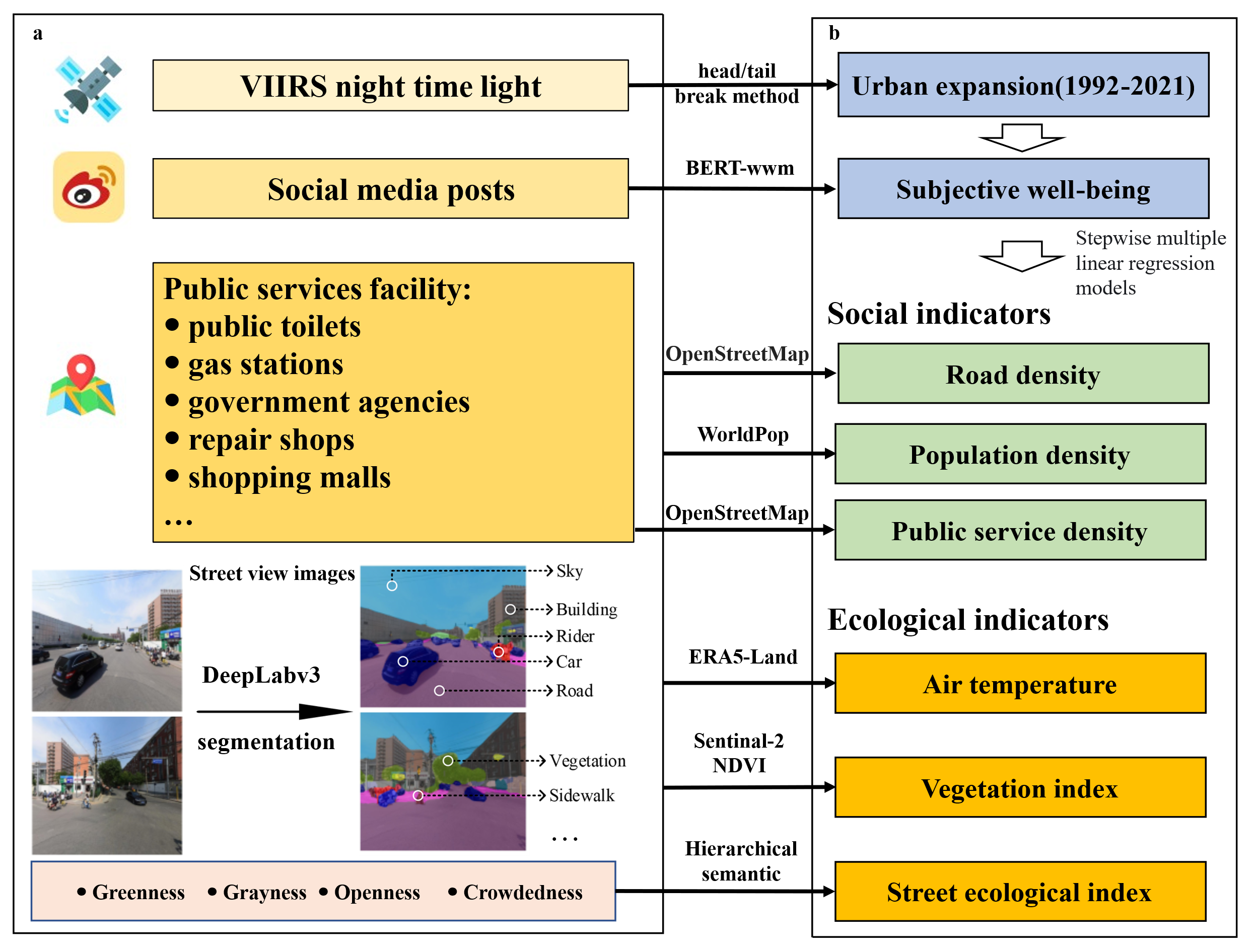


**Supplementary Figure 15.** Data and Methodology. (a) Spatial data sources, including night time light images, social media posts, public service facilities, and street view images in 107 selected cities areas. (b) The models of subjective well-being (SWB) constructed based on social and ecological indicators.


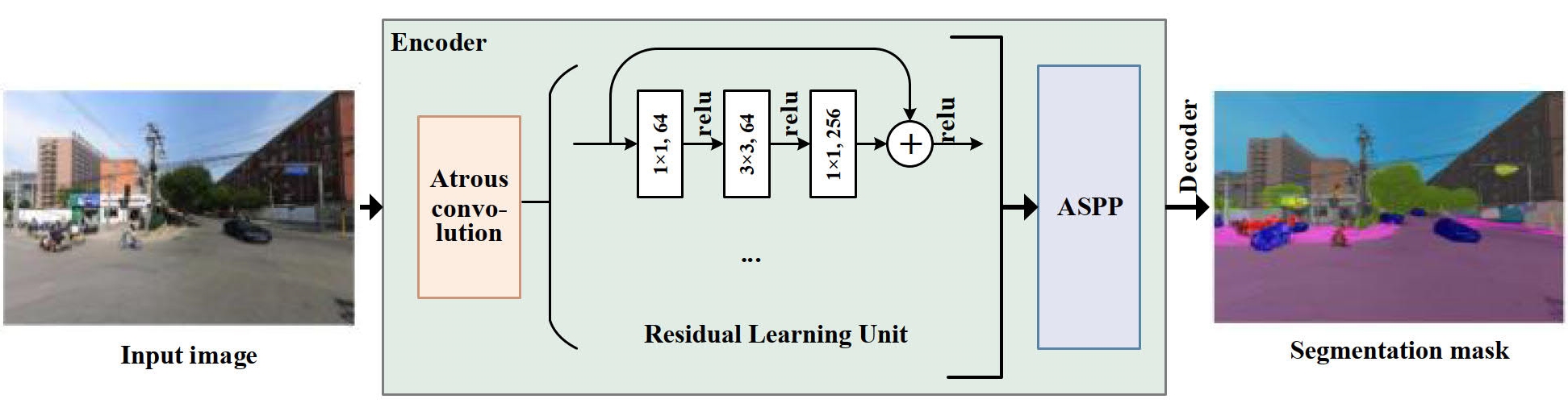


**Supplementary Figure 16.** Architecture of DeepLabv3. ASPP: atrous spatial pyramid pooling.
